# Aqueous and Ethanol Extracts of *Acacia sieberiana* (Fabaceae) Stem Bark Reverse the Pain–Depression Dyad in Mice Through Modulation of Catecholamines, Proinflammatory Cytokines, and Oxidative Stress

**DOI:** 10.1155/adpp/1244498

**Published:** 2025-02-28

**Authors:** Sorelle Ngassam Mbankou, Aliance Romain Fokoua, Cedric Wamba Koho, Roger Hermann Sadie Foguieng, Sahar Mofidi Tabatabaei, Pamela Arielle Nono Nankam, Kevin Joseph Tidgewell, Télesphore Benoît Nguelefack

**Affiliations:** ^1^Research Unit of Animal Physiology and Phytopharmacology, Faculty of Sciences, University of DSchang, Dschang, Cameroon; ^2^Pharmaceutical Sciences Department, University of Kentucky, Lexington, Kentucky 40506, USA

**Keywords:** *Acacia sieberiana*, catecholamine, cytokines, depression, oxidative stress, pain

## Abstract

**Rationale and Objective:** The pain–depression dyad is highly prevalent and has reciprocal psychological and behavioral effects, leading to poor quality of life, increased disability, and challenging therapeutic outcomes. In an attempt to find better substances that can target pain–depression comorbidity, we examined the effect of aqueous (AE) and ethanol (EE) extracts from *Acacia sieberiana* (*A. sieberiana*) stem bark on reserpinized mice (female and male Swiss albino mice aged 2-3 months).

**Methods:** The dyad was induced with 3 injections (Days 1–3) of reserpine (1 mg/kg/day, *s.c*.). Then, animals were treated (Days 4–8) with plant extracts (25, 50 and 100 mg/kg/day, *p.o*.) or L-tryptophane (100 mg/kg/day, *i.p*.). Pain-like (tactile and cold allodynia) and depression-like (pole, tail suspension, and force swimming tests) behavioral parameters were evaluated on Days 4 and 8. On Day 9, animals were sacrificed for the quantification of acetylcholinesterase activity, oxidative stress parameters, total catecholamines, dopamine, serotonin, IL-1β, and TNF-α levels in the brain or spinal cord. IL-1β and TNF-α were also assayed in the serum. The acute toxicity and phytochemical analysis of EE were conducted.

**Results:** Reserpine-induced tactile and cold allodynia, depression-like behavior, increased serum IL-1β and TNF-α, brain acetylcholinesterase activity, and decreased catecholamine concentration were all reversed by AE and EE. Plant extracts significantly increased dopamine levels and reduced oxidative stress in the brain and/or spinal cord. No significant effect was observed on brain serotonin and TNF-α. EE elicited the best pharmacological activity and was nontoxic. LC–MS/MS molecular networking phytochemical analysis identified 5 compounds with high certainty including piperine, aurantiamide acetate, and asperphenamate.

**Conclusion:** AE and EE are effective against pain and depression. Their pharmacological activities might be related to the modulation of inflammation, oxidative stress and catecholamine, and the presence of bioactive natural products.

## 1. Introduction

Pain is an unpleasant sensory and emotional experience associated with or resembling that associated with actual or potential tissue damage [1]. It is a major public health problem that negatively affects patients' quality of life. The global prevalence of unspecified pain in adults is estimated to be 27.5% [2]. A large number of literature references have largely demonstrated the sex difference in pain prevalence, with women being almost twice as affected as men [3, 4]. Pain is usually associated with psychiatric comorbidities such as depression, one of the most widespread mental illnesses worldwide. Depression is a multifactorial neuropsychiatric condition characterized by feelings of sadness, void sensation, anhedonia, sleep and appetite disturbances, and physical and cognitive changes lasting at least 2 weeks [5–7]. It affects 33.7% of the world's general population [8], and it is 50% more prevalent in women than in men [9, 10]. Biological and physiological factors, including genetics and phenotypic neural, psychological, and hormonal as well as social and cultural features, may contribute to sex differences in pain [11] and depression [12]. The two pathologies coexist in almost 80% of patients [13], and their relationship is bidirectional, as they can induce each other. In fact, it has been reported that chronic pain often induces depression, and 85% of patients reporting chronic pain issues also suffer from severe depression [14–16], while over 75% of depressed patients manifest painful physical symptoms [17]. These disorders significantly impair a person's ability to perform daily tasks and psychosocial functions [18].

To elucidate the comorbidity of pain and depression, relevant studies have demonstrated that patients with chronic pain have consistently been found to have lower levels of serotonin and low levels of norepinephrine and dopamine in plasma [19]. In addition, it has been reported that chronic pain can autonomously contribute to the pathogenesis of depression by increasing the level/expression of proinflammatory cytokines such as IL-1β, IL-6, and tumor necrosis factor α (TNF-α) [20]. Indeed, cumulative evidence from preclinical and clinical studies suggests that increases in peripheral blood inflammatory biomarkers [21] and neuroinflammation are key factors that trigger the neurobiological correlates of depression, including depletion of brain serotonin, dysregulation of the hypothalamus–pituitary–adrenal (HPA) axis, and neuronal degeneration in the hippocampus [22, 23]. In addition, the oxidative stress that accompanies pain and depression activates glial cells, which release proinflammatory cytokines (TNF-α and IL-1β), chemokines, and neurotoxic substances such as NO in the central nervous system (CNS) [24, 25]. This further amplifies the decrease in plasma tryptophan and brain serotonin to initiate or maintain depression [26, 27]. Moreover, previous studies have shown that the direct activation of cholinergic receptors or extension of endogenous acetylcholine action via pharmacological blockade of acetylcholine esterase reduces pain in rodents as well as in humans [28–30]. On the other hand, the authors in [31] showed a significant increase in acetylcholinesterase (AChE) activity in the brains of mice with depressive phenotypes and a positive correlation between AChE and superoxide anion (O_2_^•−^) levels associated with depressive behaviors.

It is obvious that pain and depression share not only comorbidities but also a number of physiopathological pathways and neurotransmitters. This fact would have normally facilitated the management of the comorbidity. However, the management of the comorbidity of pain remains challenging because the treatment should modify physiological, behavioral, and psychological processes that maintain the vicious cycle between pain and depression. Moreover, the sex difference in pain and depression is not limited only to the prevalence. It also involves the response to treatment. It has been shown that females respond to serotonergic antidepressants better than males [32]. Gender differences in pharmacological therapy and nonpharmacological pain interventions have also been reported [3]. Therefore, pain–depression management requires the search for new adequate and efficient medications that also consider gender. Plants seem to be good alternatives because they are rich in a variety of secondary metabolites with numerous pharmacological properties.

*Acacia sieberiana (A. sieberiana*) is a plant used empirically in the African pharmacopeia as rheumatic and joint pains' medicine, anti-inflammatory, antidiarrheal, and against stomachache, syphilis, liver disease, fever, tiredness, epilepsy, and mental ailments [33–35]. Pharmacological studies have shown that *A. sieberiana* has anti-hepatotoxic effects in rats [36] and antidiarrheal [37], antibacterial [33] and anticholinesterase activities [38]. Phytochemical studies have shown the presence of secondary metabolites such as saponins, tannins, cardiac glycosides, steroidal rings, resins, triterpenes, flavonoids, alkaloids and carbohydrates [35, 36]. Taken together, and given the actual traditional usage of *A. sieberiana* against pain, inflammation and mental ailment, we hypothesized that extract from *A. sieberiana* might be effective in the management of the pain–depression dyad. Consequently, the present work was undertaken to assess the therapeutic effect of the aqueous (AE) and ethanol (EE) extracts of this plant using a mouse model of pain–depression induced by reserpine. Attempts were made to evaluate the sex difference in the therapeutic effect of the plant extracts as well as the acute toxicity of EE.

## 2. Materials and Methods

### 2.1. Chemicals

Reserpine was purchased from Merck (Germany). TNF-α and IL-1β ELISA kits were purchased from R&D Systems (Germany). A serotonin ELISA kit was purchased from Abcam (United Kingdom). L-tryptophan (Cat# Art-NR 1739.1), tetramethyl benzidine, silver nitrate (Cat# Art-Nr.7908.1), sodium carbonate (Cat# BDH92284-500G), potassium carbonate (Cat# BDH9256-500G), tris buffer (Cat# Art-Nr 0188.4), potassium dichromate (Cat# 7778-50-9-500G), and sodium acetate trihydrate (Cat# 6131-90-4-1KG) were purchased from ROTH (Germany). 2,4-Dinitrophenylhydrazine (DNP) hydrochloride (Cat# EC No 259-888-5) was purchased from Tokyo Chemical Industry (United States of America). Acetylthiocholine (A5751-5G), potassium periodate (PPI) (Cat# 210056-100G), trichloroacetic acid (Cat# T6399-100G), orthophosphoric acid (Cat# 7664-38-2), thiobarbituric acid (Cat# T5500-100G), adrenaline (Cat# E4250-5G), hydrogen peroxide (Cat# Art-Nr 9681.1), glacial acetic acid (Cat# 64-19-7), 5,5′-dithiobis-2-nitrobenzoic acid (Cat# D8130-10G), trisodium citrate acid (Cat# 68-04-2), naphtylethylene diamine dihydrochloride (Cat# 222488-25G), sulfanilamide (Cat# S9251-500G), and sodium nitrate (Cat# 237213-5G) were purchased from Sigma-Aldrich (Germany).

### 2.2. Animals

Female and male Swiss albino mice (20–30 g), aged 2-3 months, were obtained from the animal facility of the Research Unit of the Neuroinflammatory and Cardiovascular Pharmacology of the Department of Animal Biology at the University of Dschang. They were housed in plastic cages in groups of 5 with food and water available *ad libitum*. The temperature was controlled at 24 ± 2°C, humidity at 50%–60%, and an approximately 12:12 h light-dark cycle. Mice were grouped and acclimated to the laboratory conditions for at least 2 weeks before the start of the experiment. All animal procedures were performed in agreement with the ethical rules of animal care described by the European Committee Guidelines 86/609/EEC and validated by the department committee (Department of Animal Biology, Faculty of Science 025/13/304FSa). The sample size was calculated using the E method. Cages and animals were labeled to avoid confounders.

### 2.3. Plant Material, Extraction, and Phytochemical Profiling

#### 2.3.1. Extraction

*A. sieberiana* materials were collected in Meiganga in the Mbéré division, Adamaoua Region, Cameroon, in January 2019 and used for the identification of the plant at the national herbarium where a voucher specimen was deposited under reference 58943/HNC. Fresh barks were shade-dried and ground for extract preparation. Aqueous extraction was performed by decoction of 125 g of powder in 1 L of distilled water for 6 h at 60°C. The resulting filtrate was dried, weighed, and stored in the dark at −2°C. The extraction yield was 5.52%. The EE was obtained by macerating 625 g of powder in 5 L of ethanol for 48 h with occasional stirring. The filtrate obtained was then concentrated in a rotary evaporator, with an extraction yield of 3.65%.

#### 2.3.2. Phytochemical Profiling

LC–MS/MS molecular networking was conducted using the GNPS2 platform [39, 40]. In brief, chromatography was performed on a Shimadzu DGU-20A 3R system coupled to a SCIEXT Q-TOF 56000 mass spectrometer and equipped with a dual spray electrospray ionization source. MS data were acquired in both positive and negative ion modes over the 100–1200 m/z range using a survey scan method allowing simultaneous acquisition of MS and MS/MS data for the sample. The sample (EE) was prepared in methanol at 3 mg/mL and 20 μL was injected for each LC–MS/MS analysis run. Molecular networking and data visualization were performed using the online libraries from the GNPS2 website (https://gnps2.org). A summary of the spectral data is in the Supporting file and publicly available on GNPS2 using the Task ID numbers.

### 2.4. Experimental Protocol

The doses of the extracts used in this study were obtained from the combination of traditional practices and our preliminary studies. The dose of L-tryptophan was chosen based on data from the literature [41]. The induction of the pain–depression dyad was performed using the protocol described by [42] with slight modifications.

**Figure 1:**
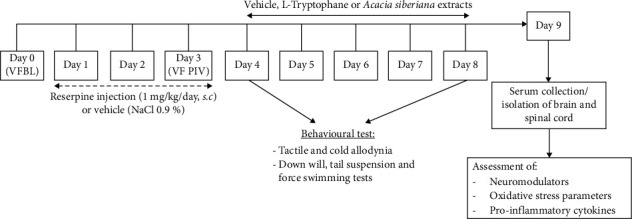
Experimental design to assess the effects of *Acacia sieberiana* extracts on pain–depression dyad induced by reserpine in mice.

The baseline reaction to von Frey hair was measured in 100 mice (sex ratio) on Day 0, and 90 of them were selected based on their baseline reaction and divided into two sets, A (10 mice) and B (80 mice). The mice in Sets A and B received subcutaneous injections of normal saline with 1% DMSO or reserpine (at a final dose of 1 mg/kg, dissolved in 1% DMSO) respectively, for three consecutive days (Days 1–3). On Day 4, a second baseline reaction in the von Frey test was taken. Set A constituted the naïve group (Group 1) and was treated daily with distilled water (extracts' vehicle), while Set B was subdivided using stratified randomization into 8 groups of 10 animals each (5 females and 5 males) and treated as follows: Group 2 (disease control) received distilled water; Group 3 (reference) received L-tryptophan (100 mg/kg/d, *i.p*.); Groups 4, 5, and 6 were treated orally with AE at doses of 25, 50, and 100 mg/kg, respectively; and groups 7, 8, and 9 received EE orally at doses of 25, 50, and 100 mg/kg. Treatments were given on a daily basis for 5 consecutive days (Days 4–8) (Figure 1). All pain-like (tactile and cold allodynia) and depression-like (pole test, forced swimming test [FST], and tail suspension test [TSTs]) behavioral tests were performed on Day 4 to test the effect of a single administration and on Day 8 to evaluate the effect of repeated administration of treatments (Figure 1).

On Day 9, animals were anesthetized with diazepam (1 mg/kg) and ketamine (5 mg/kg) via intraperitoneal injections. Blood samples were collected through the retro-orbital sinus with capillary tubes. The collected blood was centrifuged at 3000 rpm, and the serum obtained was stored at −80°C for the cytokine assay. After blood collection, mice were rapidly dissected, brain and spinal cord were removed and immediately homogenized (10%, w/v) in ice-cold Tris buffer (10 mM, pH 7.4), cold (4°C) centrifuged for 15 min at 10,000 rpm, and the supernatants were collected and stored at −80°C. The supernatant was used for the determination of nitric oxide (NO), malondialdehyde (MDA), superoxide dismutase (SOD), and glutathione (GSH). The catalase (CAT), catecholamines, dopamine, serotonin and AChE contents or activities were estimated only in the brain.

To evaluate the sex-related difference in treatment response in all parameters, we calculated the percentage change of the effect versus the control group according to the equation as follows:

(1) $$\begin{array}{c} \%\text{ change}=\frac{Vc-Vt}{Vc}\times 100\text{ or }\frac{Vt-Vc}{Vc}\times 100,\text{depending on the direction of variation}, \end{array}$$

where Vc is the value of the control group and Vt is the value of the test group.

The acute toxicity study was performed according to the WHO [43] protocol using the most active extract.

### 2.5. Pain-Like Behavioral Tests

To assess the antinociceptive potentials of the plant extracts, two protocols were used: the tactile allodynia test, which assesses mechanical pain sensation, and the acetone test, which measures cold allodynia. These behavioral experiments were performed by trained evaluators in blinded protocols.

#### 2.5.1. Tactile Allodynia

This test was performed according to the protocol previously described by [44]. Mice were placed in individual plastic cages with floor metal mesh and acclimated for 1 h. Tactile allodynia was determined by assessing the 50% left hind paw withdrawal threshold (PWT) to tactile stimuli using a series of von Frey filaments of increasing forces (Stoelting Co., Wood Dale, Illinois, United States of America) through the up and down method. In line with this paradigm and considering mice as animal subjects, testing series were initiated with the 0.008 g hair and whether ascending or descending, stimuli were always presented in a consecutive fashion. As such, the immediate stronger stimulus was presented in the absence of a paw withdrawal response to the initially selected hair. In the event of paw withdrawal, the next weaker stimulus was chosen. The test was stopped when one hair filament and its immediate stronger filaments induced negative and positive responses, respectively, in two consecutive testing. A positive response was defined as an immediate withdrawal of the hind paw. The Chaplan table was used to determine the 50% withdrawal threshold as described by Chaplan et al. [44]. According to Dixon's method, the optimal threshold calculation by this method requires 6 responses in the immediate vicinity of the 50% threshold. Thus, the pattern used for calculation was made of 6–9 responses, where *X* refers to withdrawal (positive response) and 0 refers to no withdrawal (negative response). The 50% response threshold was interpolated using the following formula:

(2) $$\begin{array}{c} 50\%\;g\text{ threshold}=\frac{10^{\left[Xf+k\delta \right]}}{10,000}, \end{array}$$

where *Xf* is the value (in log units) of the final von Frey hair used, *k* is the tabular value for the pattern of positive/negative responses, and *δ* is the mean difference (in log units) between stimuli (here, it is 0.224). This test was performed 1 hour after the oral administration of the plant extracts.

#### 2.5.2. Cold Allodynia

The acetone test measures aversive behaviors triggered by cooling generated by the evaporation of a drop of acetone applied to the medial area of the hind paw and is considered a measure of cold allodynia [45]. Acetone (50 μL) was applied to the left paw of each animal. The outcome measured was the time the animal spent lifting, licking, flicking, or biting the left hind paw after each application. This test was performed 2 h after the oral administration of plant extracts.

### 2.6. Depression-Like Behavioral Tests

Three tests were used to evaluate the antidepression effect of EE. The three experiments (pole test, TST, and FST) were video recorded, and the videos were analyzed with Any-maze software to generate row data.

#### 2.6.1. Pole Test

To assess the depression-like behavior in mice, we performed the pole test using a modified method of [46]. The procedure evaluates the ability of a mouse to grasp and maneuver on a pole to descend to its flat surface. A vertical steel pole with a rough surface (55 cm high and 1 cm in diameter) was placed in the home cage. Mice were placed at 5 cm to the top of the pole with their heads facing upward. The time spent orienting down (t-turn) and descending the pole (t-descend) was measured over a 5-min period. The animal was given the maximal time (5 min) if he failed to turn or to move down the pole. This test was performed 3 h after oral administration of the plant extracts.

#### 2.6.2. TST

The TST is widely used to assess antidepressant activity in mice. It is based on the fact that animals subjected to inescapable short-term stress, namely, being suspended by the tail, develop an immobile posture. The total duration of immobility throughout the TST was measured as described by Can et al. [47]. In brief, mice were suspended 55 cm above the ground with tape placed approximately 2 cm from the tip of their tail. After 2 min of acclimation, the test was recorded for a period of 4 min. Mice were considered immobile only when they were passively suspended and completely immobile. This test was performed 4 h after oral administration of the plant extracts.

#### 2.6.3. FST

The FST was conducted as described by [48]. This test was performed on mice, who were individually forced to swim in an open container (width 20 cm, height 30 cm) containing 15 cm of water at 26 ± 1°C. After 2 min of acclimatization, the immobility time was recorded for 5 min. Immobility time was the time spent by the mice floating in the water without struggling, making only movements necessary to keep the head above water. A decrease in immobility time indicated a reduction in depression-related behavior. The test was carried out 5 h after treatment with the plant extracts.

### 2.7. Assay of Biochemical Parameters

To understand the mechanisms by which AE and EE could induce behavioral changes in animals, a number of biochemical parameters that are affected during pain and depression conditions were assayed, including total catecholamine, dopamine, serotonin, inflammatory cytokines (IL-1β and TNF-α), AChE activity, and oxidative stress.

#### 2.7.1. Total Catecholamine Assay

Brain's total catecholamine content was assessed using the protocol described by [49] with minor modifications. In the reaction medium, 100 μL of TMB solution (1 mM), 50 μL of AgNO_3_ solution (3 mM), 340 μL of NaAc buffer (0.2 M, pH 5.0), and 10 μL of homogenate or catecholamine were sequentially added. Subsequently, the mixture was incubated for 5 min at room temperature, and the absorbance was measured at 652 nm on a spectrophotometer. The concentration of catecholamine was determined using the dopamine standard curve.

#### 2.7.2. Determination of the Dopamine Level

Dopamine is the principal catecholamine that at a low level is associated with depressive disorder. The measurement of dopamine levels in the brain was performed according to the method described by [50] with slight modifications. The method is based on the coupling of dopamine with a diazonium salt to produce an intensely colored azo derivative. DNP was oxidized with PPI to produce a diazonium salt that was coupled with dopamine in a basic medium. Thus, to 100 μL of DNP (0.5 mM in sulfuric acid), 100 μL of PPI (0.65 mM in distilled water), 50 μL of NaOH (1 M, in distilled water), and 10 μL of supernatant or dopamine were added. The mixture was vortexed, and the absorbance was measured at 560 nm against a blank. The concentration of dopamine was determined based on a dopamine standard curve.

#### 2.7.3. Assay of Oxidative Stress Parameters

To assess the oxidative status of the animals after treatment, brain and spinal cord homogenates were used. MDA was assessed using the method described by [51]. The SOD activity assay was measured according to the protocol described by [52]. The method of Sinha [53] was used to determine the CAT activity in the brain. The GSH concentration was measured as described by [54]. The NO assay was conducted as described by [55] using the Griess reagent. The concentration of nitrite in the tissue homogenates was calculated using a NaNO_2_ standard curve.

#### 2.7.4. Estimation of Serotonin, TNF-α, and IL-1β

Serotonin concentrations in the brain (Abcam) and TNF-α and IL-1β levels (R&D Systems) in the serum and brain of mice were determined using commercial ELISA kits following the manufacturer's instructions.

#### 2.7.5. Determination of AChE Activity

The determination of AChE was performed according to the method of [56] with some modifications. In brief, 100 μL of DTNB (0.01 M in Tris 0.1 M, pH 7.4) was mixed with 100 μL of the supernatant. The reaction was started by adding 50 μL of acetylthiocholine iodide (0.05 M). The absorbance was measured at 405 nm every minute for 3 min against a blank. Enzyme activity was calculated using the extinction coefficient = 1.36 ⨯ 10^4^.

### 2.8. Acute Toxicity Assessment

The acute toxicity study was performed according to the protocol in [43]. Prior to the experiment, animals were checked for well-being and fasted for 12 h. They were further divided into five groups of 10 mice (5 males and 5 females) treated as follows: the control group was treated with distilled water, and the other four groups received a single administration of EE at doses of 200, 600, 1800 and 5400 mg/kg. The doses were selected as multiples of the effective dose, with one above the high limit of nontoxic (5000 mg/kg) fixed by WHO. Animals were then observed for 1 h after administration for any behavioral changes. The locomotor activity of each animal was investigated at 1 h postadministration using the open field test [57, 58]. They were video recorded and analyzed using ANY-maze software. Subsequently, the mortality, body mass, fecal status, and general appearance of the mice were recorded once a day for 14 days.

### 2.9. Statistical Analysis

The results are expressed as the mean ± standard error of the mean (SEM). Statistical analyses were performed using GraphPad Prism software Version 8.4.2. The variance homogeneity was assessed by Spearmann's rank correlation. The normality of data was tested using Shapiro–Wilk and Kolmogorov–Smirnov tests. Where data were not normally distributed, a log transformation was performed. Intergroup variation was measured with one-way analysis of variance (ANOVA) followed by Dunnett's posttest or two-way repeated-measures ANOVA followed by Dunnett's multiple comparison posttest. For longitudinal data, we assumed no sphericity and directly applied Greenhouse–Geisser correction. For the sex-related response to treatment, data from all treated animals were normalized as a percentage of their respective sex in the disease control group. For repeated measurements, the area under the curve (AUC) was used for extract or sex comparison. The multiple *t*-test (Holm–Sidak method) was used to analyze differences between the two extracts or the two sexes at the same dose of the extracts. No data points were excluded during analyses. Statistical significance was considered at *p* < 0.05.

## 3. Results

### 3.1. Phytochemical Profiling

**Figure 2:**
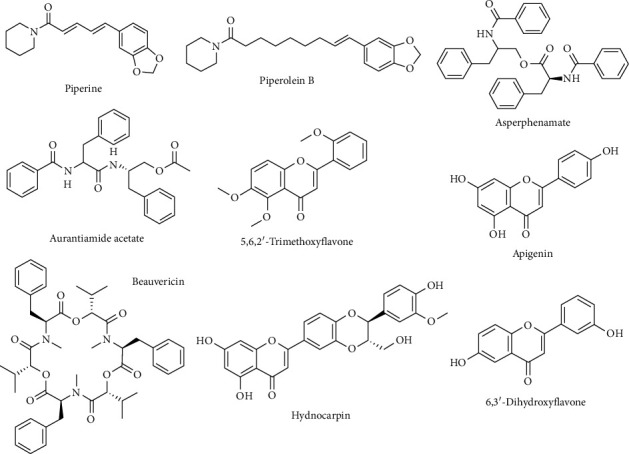
Compounds identified in the ethanol extract of stem bark of *Acacia sieberiana* through LC–MS/MS molecular networking.

The phytochemical analysis was conducted using LC–MS/MS molecular networking of the ethanol extract of *Acacia sieberiana*. Analysis of the extract shows strong evidence for the presence of 5 compounds, including alkaloids (piperine and piperolein B), carboxylic esters (asperphenamate and aurantiamide acetate), and an enniatin compound beauvericin (Supporting file (available here)). There is also evidence for the presence of a number of flavonoids (5,6,2′-trimethoxyflavone, apigenin, hydnocarpin, and 6,3′-dihydroxyflavone) (supporting file (available here)); however, since this was a bark extract, they are in low amounts and so their presence is not absolutely verified (Figure 2).

### 3.2. *A. sieberiana* Extracts Reduced Tactile Allodynia

**Figure 3:**
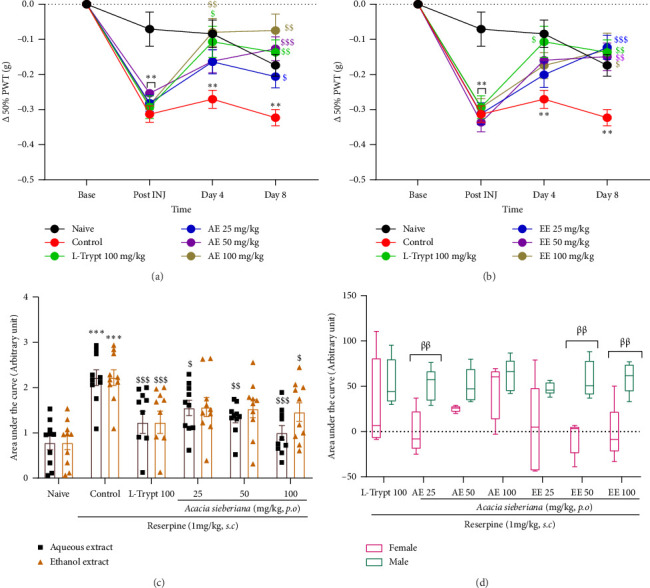
Aqueous and ethanol extracts of *A. sieberiana* stem bark reversed tactile allodynia measured with von Frey hairs in reserpinized mice (a) and (b) Δ 50% PWT (g) of mice in different groups during 8 days of the experiment for AE and EE, respectively, (c) area under the curve representing the difference between the two extracts at the same dose, and (d) sex effect of percentage change in VFT from the control group. *n* = 10 (5 females + 5 males). ⁣^∗∗^*p* < 0.01 and ⁣^∗∗∗^*p* < 0.001 show a significant difference compared to the naïve group. ^$^*p* < 0.05, ^$$^*p* < 0.01, and ^$$$^*p* < 0.001 show a significant difference compared to the control group (two-way ANOVA with Dunnett's multiple comparisons posttest). ^ββ^*p* < 0.01 shows a significant difference between females and males at the same dose (multiple *t*-test and Holm–Sidak method). AE: aqueous extract; EE: ethanol extract; L-trypt: L-tryptophan; PWT: pain withdrawal threshold.

Reserpine injection into mice resulted in a significant increase in tactile allodynia compared to that in the naïve group. The administration of AE and EE significantly reversed this tactile allodynia on Day 4 at the highest doses for AE (69.14% reversion) and on Day 8 of treatment at all doses of both extracts. Maximal reductions of 76.90% and 62.00% in allodynia were observed with AE at a dose of 100 mg/kg and EE at 25 mg/kg, respectively (Figures 3(a) and 3(b)). The AUC showed no significant difference between the effects of the two extracts (Figure 3(c)). As presented in Figure 3(d), male mice showed a better response to the analgesic effect of both AE and EE. Significant differences (*p* < 0.01) were observed with AE at the dose of 25 mg/kg and EE at the doses of 50 and 100 mg/kg.

### 3.3. *A. sieberiana* Extracts Reversed Cold Allodynia

**Figure 4:**
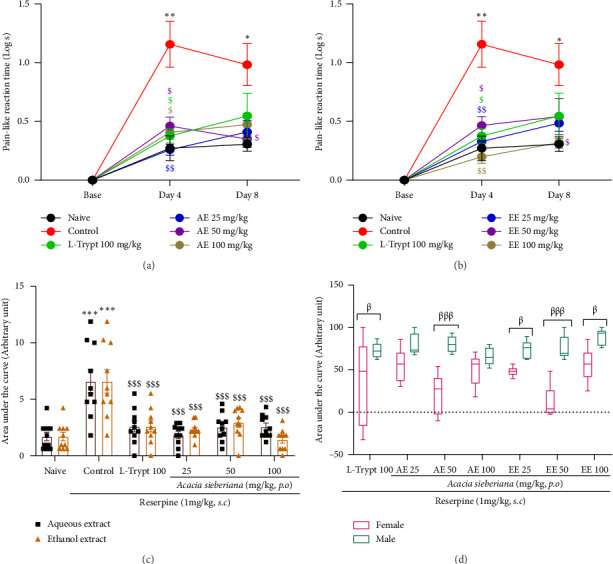
Aqueous and ethanol extracts of *A. sieberiana* stem bark reversed acetone-induced cold allodynia in reserpinized mice: (a and b) pain-like reaction time of mice in different groups during 8 days of the experiment for AE and EE, respectively, (c) area under the curve representing the difference between the two extracts at the same dose, and (d) sex-dependent response to plant extract treatment. *n* = 10 (5 females + 5 males). ⁣^∗^*p* < 0.05, ⁣^∗∗^*p* < 0.01, and ⁣^∗∗∗^*p* < 0.001 show a significant difference compared to the naïve group. ^$^*p* < 0.05, ^$$^*p* < 0.01, and ^$$$^*p* < 0.001 show a significant difference compared to the control group (one-way ANOVA with Dunnett multiple comparisons posttest). ^β^*p* < 0.05, ^ββ^*p* < 0.01, and ^βββ^*p* < 0.001 indicate significant differences between females and males at the same dose of the same extract (multiple *t*-test and Holm–Sidak method). AE: aqueous extract; EE: ethanol extract; L-trypt: L-tryptophan.

Animals from the control group developed cold allodynia after the injection of reserpine. The reaction time was significantly high on Days 4 (*p* < 0.01) and 8 (*p* < 0.05) of the experimental period. The single administration (Day 4) or repeated treatment (Day 8) with both AE and EE significantly reversed the reserpine-induced allodynia (Figures 4(a) and 4(b)). The global analysis using the AUC showed a significant (*p* < 0.001) effect of both extracts at all the doses used, with no significant difference between the two extracts (Figure 4(c)). However, male mice proved to be highly sensitive (*p* < 0.05 to *p* < 0.001) to the treatment compared to females (Figure 4(d)).

### 3.4. *A. sieberiana* Extracts Mitigated Depression-Like Behavior in the Pole Test

**Figure 5:**
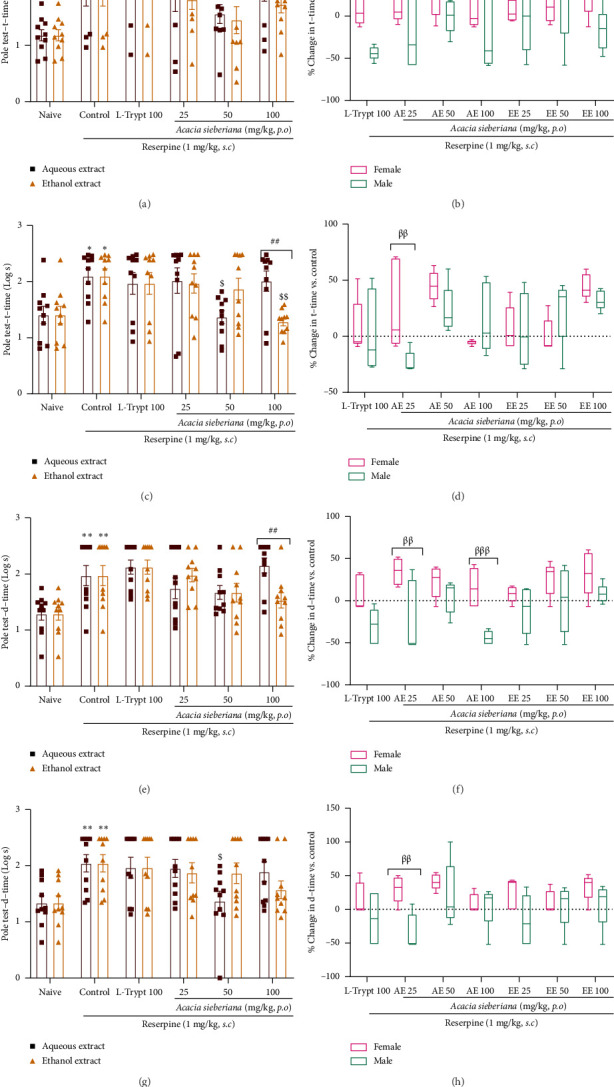
Aqueous and ethanol extracts of *A. sieberiana* stem bark reduced turn and descending times in reserpinized mice: (a and c) turn time of mice in different groups at 4 and 8 days of the experiment, (e and g) descent time of mice at 4 and 8 days of the experiment, (b and d) sex effect on the percentage change in turn time compared to the control, and (f and h) sex effect percentage change in descent time to control. *n* = 10 (5 females + 5 males). ⁣^∗^*p* < 0.05 and ⁣^∗∗^*p* < 0.01 show a significant difference compared to the naïve group. ^$^*p* < 0.05 and ^$$^*p* < 0.01 show a significant difference compared to the control group (one-way ANOVA with Dunnett's multiple comparisons posttest). ^##^*p* < 0.01 shows a significant difference between the two extracts at the same dose (multiple *t*-test and Holm–Sidak method). ^β^*p* < 0.05, ^ββ^*p* < 0.01, and ^βββ^*p* < 0.001 indicate significant differences between females and males at the same dose (multiple *t*-test and Holm–Sidak method). AE: aqueous extract; EE: ethanol extract; L-trypt: L-tryptophan; t-time: turn time; d-time: descending time.

The pole test was performed on Days 4 and 8 of the experimental period to evaluate the antidepressant effects of the plant extracts. As depicted in Figure 5, reserpine treatment significantly increased the t-time (turn time on the bar) (*p* < 0.05) and the d-time (descend time on the bar) compared to the naïve group. The single administration (Day 4) of AE and EE failed to reduce the effect of reserpine. However, a significant difference (*p* < 0.01) was observed between the effects of AE and EE at a dose of 100 mg/kg (Figures 5(a) and 5(e)).

After repeated treatment with the plant extracts, both extracts reduced the t-time, but the effect of AE was not dose-dependent. AE (50 mg/kg) and EE (100 mg/kg) significantly reduced the turn time by 34.83% (*p* < 0.05) and 38.86% (*p* < 0.01), respectively, when compared to the control (Figure 5(c)). Only AE at a dose of 50 mg/kg significantly (*p* < 0.05) reduced the d-time by 33.09% (Figure 5(g)).

In general, females showed the best responses to the treatment both after a single treatment and repeated administration (Figures 5(b), 5(d), 5(f), and 5(h)).

### 3.5. *A. sieberiana* Reduced Immobility in TST But Not in FST

**Figure 6:**
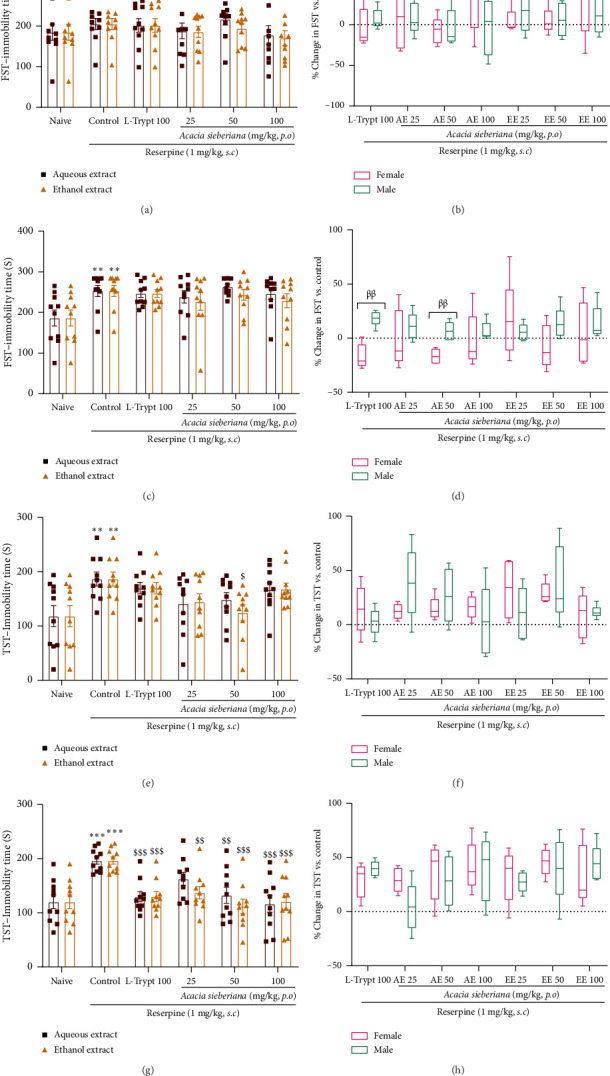
Aqueous and ethanol extracts of *A. sieberiana* stem bark reduced immobility time in the TST but not in the FST in reserpinized mice: (a and c) FST immobility time in different groups on Days 4 and 8 of the experimental period, (e and g) TST immobility time in different groups of mice on Days 4 and 8 of the experiment, respectively, (b and d) sex-dependent response in the FST and (f and h) sex-dependent response in the TST. *n* = 10 (5 females + 5 males). ⁣^∗∗^*p* < 0.01 and ⁣^∗∗∗^*p* < 0.001 show a significant difference compared to the naïve group. ^$^*p* < 0.05, ^$$^*p* < 0.01, and ^$$$^*p* < 0.001 show a significant difference compared to one-way ANOVA with Dunnett's multiple comparisons posttest. AE: aqueous extract; EE: ethanol extract; L-trypt: L-tryptophan; FST: forced swim test; TST: tail suspension test.

In the FST, immobility was assessed as a sign of depression in animals treated with reserpine alone or with plant extracts. The results are reported in Figure 6. Reserpine injection induced a significant (*p* < 0.01) increase in immobility time over the 8 days of the experiment. Neither the single administration nor the repeated treatment affected the evaluated parameter (Figures 6(a) and 6(c)).

With regard to the TST, the immobility time in reserpine-treated animals was significantly higher than that in naïve animals on Days 4 (*p* < 0.01) and 8 (*p* < 0.001) (Figures 6(e) and 6(g)). This was, however, significantly reduced by EE at a dose of 50 mg/kg on Day 4 (*p* < 0.05) (Figure 6(e)) and in a dose-dependent manner by *A. sieberiana* extracts on Day 8 (*p* < 0.01; *p* < 0.001) (Figure 6(g)). No significant difference was observed between the effects of the two extracts or in the sex-dependent response to the plant extract treatment (Figures 6(f) and 6(h)).

### 3.6. *A. sieberiana* Extracts Reversed Total Catecholamine Depletion in the Brain But Not Serotonin

**Figure 7:**
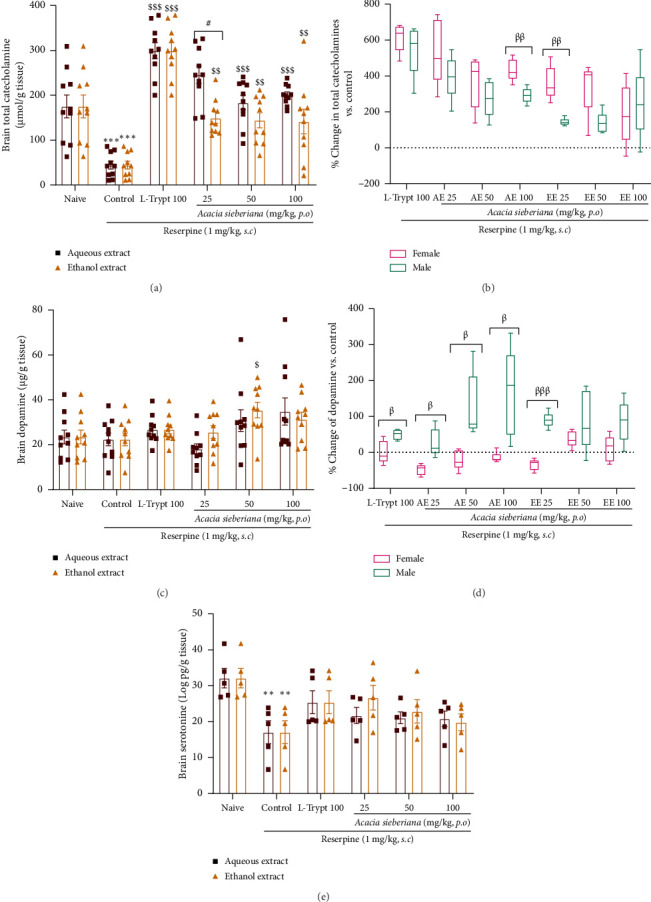
Aqueous and ethanol extracts of *A. sieberiana* stem bark reversed the drop in brain catecholamines but not serotonin content in reserpinized mice: (a) brain concentration of catecholamines after 8 days of the experiment, (c) brain concentration of dopamine after 8 days of the experiment, (b and d) sex-related response to *A. sieberiana* extracts in brain concentrations of catecholamine and dopamine, and (e) brain concentration of serotonin in females after 8 days of the experiment. *n* = 10 (5 females + 5 males). ⁣^∗∗∗^*p* < 0.001 shows a significant difference compared to the naïve group. ^$^*p* < 0.05, ^$$^*p* < 0.01, and ^$$$^*p* < 0.001 show a significant difference compared to the control group (one-way ANOVA with Dunnett's multiple comparisons posttest). ^#^*p* < 0.05 significant difference between the two extracts at the same dose (multiple *t*-test and Holm–Sidak method). ^β^*p* < 0.05, ^ββ^*p* < 0.01, and ^βββ^*p* < 0.001 indicate significant differences between females and males at the same dose (multiple *t*-test and Holm–Sidak method). AE: aqueous extract; EE: ethanol extract; L-trypt: L-tryptophan.

As depicted in Figure 7(a), reserpine administration resulted in a significant (*p* < 0.001) decrease in total catecholamine levels in the brains of mice compared to the naïve group. Interestingly, both plant extracts at all doses reversed this effect and significantly (*p* < 0.001) increased the level of total catecholamine. The maximal effect was obtained at a dose of 25 mg/kg. Sex comparison showed better activity in female mice, with a significant difference at the dose of 100 mg/kg for AE and at 25 mg/kg for EE (Figure 7(b)).

The dopamine concentration in the animals' brains did not change after treatment with reserpine. However, the dose of 50 mg/kg EE increased dopamine levels by 53.04% (*p* < 0.05) compared to the diseased control (Figure 7(c)). A strong sex difference was observed with AE at all doses tested and with EE at the dose of 25 mg/kg (Figure 7(d)).

The brain concentration of serotonin was evaluated only in females. Reserpine injection significantly (*p* < 0.01) reduced the brain's serotonin content, but plant extracts were unable to significantly reverse this effect (Figure 7(e)).

### 3.7. Effect of *A. sieberiana* Extracts on Oxidative and Nitrosative Stress

To evaluate the total oxidation level in the brain, we quantified tissue MDA levels. Compared to the naïve group, MDA levels were not significantly increased in the brain and spinal cord in reserpine-treated mice (Table 1). Nevertheless, EE significantly (*p* < 0.01) decreased MDA concentrations in the brain (dose 25 mg/kg) and spinal cord (50 mg/kg) by 27.91% and 40.37%, respectively. EE was also significantly more effective in reducing MDA than AE (Table 1).

Oxidative stress status was further assessed by measuring the activity of antioxidant enzymes in the brain and spinal cord of mice. The activity of SOD and CAT and the level of GSH did not significantly change in the brains of animals after reserpine treatment compared to the naïve group (Table 1). However, AE at a dose of 100 mg/kg significantly (*p* < 0.05) increased CAT activity by 26.55%, while GSH was significantly (*p* < 0.05) increased by AE at a dose of 50 mg/kg and with all tested doses of EE. The maximal effect of 314.66% (*p* < 0.001) was observed at a dose of EE 50 mg/kg. When comparing the effect of the two extracts, EE was more efficient than AE in increasing GSH, while at 100 mg/kg, the effect of AE was more potent than that of EE (Table 1).

In the spinal cord, SOD activity was significantly (*p* < 0.001) reduced by reserpine administration. Only mice treated with EE showed a significant increase in SOD levels, with a maximal effect (*p* < 0.001) obtained at a dose of 25 mg/kg. At this dose, EE was even more potent than AE. Notably, EE at a dose of 50 mg/kg significantly (*p* < 0.001) increased the GSH level in the spinal cord (Table 1).

NO levels were found to be significantly increased in the brain (*p* < 0.01) and spinal cord (*p* < 0.001) of reserpine-treated mice. Both extracts at all doses significantly reduced NO levels in the brain (*p* < 0.001). The same effect was observed in the spinal cord except for the dose of 25 mg/kg AE, which did not induce any effect (Table 1).

### 3.8. *A. sieberiana* Extracts Reduced Serum Cytokines But Not Brain Contents

**Figure 8:**
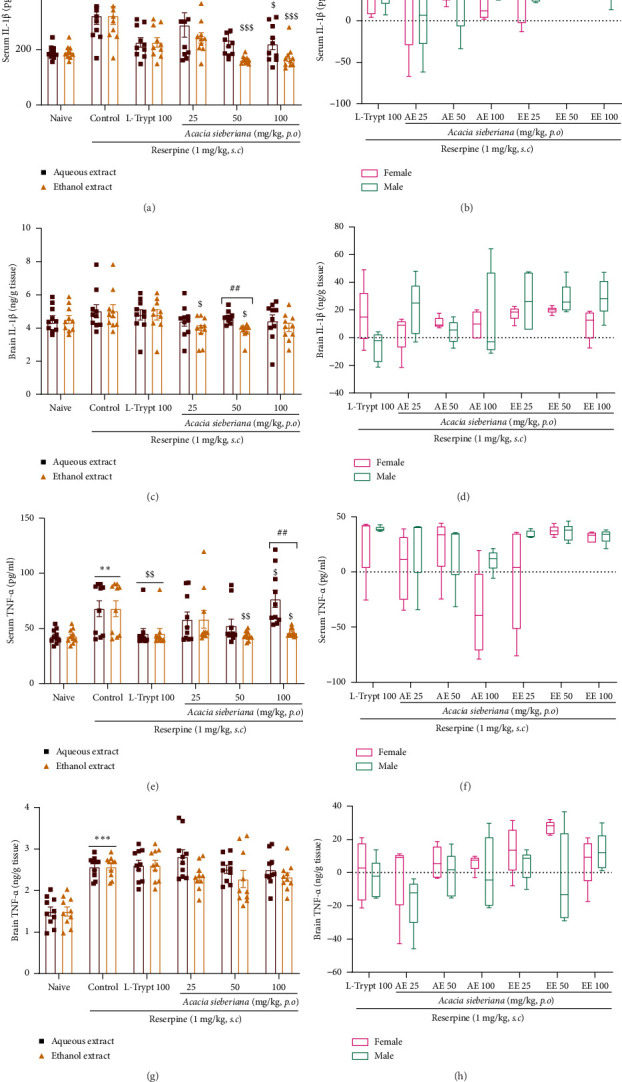
Aqueous and ethanol extracts of *A. sieberiana* stem bark reduced serum IL-1β and TNF-α levels but not brain tissue concentrations in reserpinized mice: (a and c) effects of AE and EE on the serum and brain IL-1β, (b and d) sex effect of plant extracts on serum and brain concentration of IL-1β, (e and g) effects of AE and EE on the serum and brain TNF-α, and (f and h) sex effect of *A. sieberiana* extracts on serum and brain TNFα concentrations. *n* = 10 (5 females + 5 males). ⁣^∗∗∗^*p* < 0.001 shows a significant difference compared to the naïve group. ^$^*p* < 0.05, ^$$^*p* < 0.01, and ^$$$^*p* < 0.001 show a significant difference compared to the control group (one-way ANOVA with Dunnett's multiple comparisons posttest). ^#^*p* < 0.05 and ^###^*p* < 0.01 show q significant difference between the two extracts at the same dose (multiple *t*-test and Holm–Sidak method). ^ββ^*p* < 0.01 and ^βββ^*p* < 0.001 show a significant difference between females and males at the same dose (multiple *t*-test and Holm–Sidak method). AE: aqueous extract; EE: ethanol extract; L-trypt: L-tryptophan.

To examine whether *A. sieberiana* effects are mediated through anti-inflammatory activity, we assayed cytokines in both serum and brain. The effects of *A. sieberiana* extracts on inflammatory markers are summarized in Figure 8. Treatment with reserpine significantly (*p* < 0.01) increased serum concentrations of IL-1β and TNF-α (*p* < 0.001), whereas only TNF-α concentration was elevated in the brain (*p* < 0.001) compared to the naïve group. Animals treated with *A. sieberiana* extracts showed a significant reduction in serum IL-1β and TNF-α concentrations at all doses (*p* < 0.001), except for the 100 mg/kg AE dose, which did not induce any change in TNF-α levels (Figures 8(a) and 8(e)). EE had a greater effect than EA on serum IL-1β (*p* < 0.05) and TNF-α (*p* < 0.001) at doses of 50 and 100 mg/kg, respectively (Figures 8(a) and 8(e)). In contrast to the serum samples, treatment with AE or EE did not affect these proinflammatory cytokines in the brain (Figures 8(c) and 8(g)).

Considering the sex-dependent response, there is no significant difference between males and females in response to the plant extract treatment. However, it was observed that females have a lower level of brain IL-1β than males (Figures 8(b), 8(d), 8(f), and 8(h)).

### 3.9. *A. sieberiana* Reversed Hyper AchE Activity in the Brain

**Figure 9:**
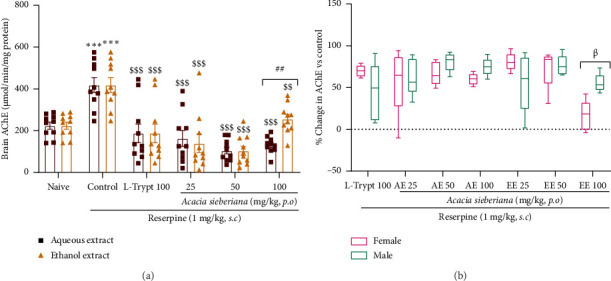
Aqueous and ethanol extracts of *Acacia sieberiana* stem bark reversed the elevated brain acetylcholinesterase induced by reserpine injection: (a) comparison of plant extract effects on brain AchE activity and (b) sex-related effect of *A. sieberiana* on brain AChE activity. *n* = 10 (females + 5 males). ⁣^∗∗∗^*p* < 0.001 shows a significant difference compared to the naïve group. ^$$^*p* < 0.01 and ^$$$^*p* < 0.001 show a significant difference compared to the control group (one-way ANOVA with Dunnett's multiple comparisons posttest). ^##^*p* < 0.01 shows a significant difference between the two extracts at the same dose (multiple *t*-test and Holm–Sidak method). ^β^*p* < 0.05 shows a significant difference between females and males at the same dose (multiple *t*-test and Holm–Sidak method). AE: aqueous extract; EE: ethanol extract; L-trypt: L-tryptophan; AChE: acetylcholinesterase.

Reserpine administration significantly (*p* < 0.001) increased AChE concentrations in mice. This increase was significantly reduced by treatment with both *A. sieberiana* extracts at all doses, with maximal effects of approximately 75% (*p* < 0.001) obtained at a dose of 50 mg/kg of both extracts (Figure 9(a)).

The sex-related effect of plant extracts showed that when treated with EE at a dose of 100 mg/kg, the plant extract was significantly (*p* < 0.05) more effective in males than in females (Figure 9(b)).

### 3.10. *A. sieberiana* Ethanol Extract Has Relatively Low Toxicity in Mice

**Figure 10:**
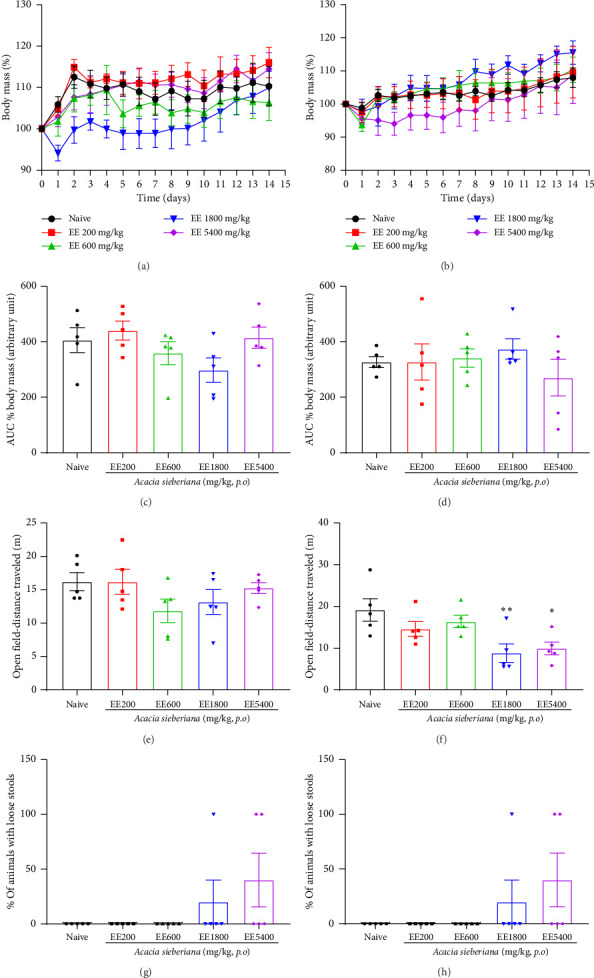
Acute toxicity tests of ethanol extracts of *A. sieberiana* stem bark in mice: (a and b) time-dependent variation in body mass percentage in female and male mice, (c and d) area under the curve (AUC) of the body mass variation in female and male mice during 14 days after unique oral administration of EE, (e and f) distance traveled in open field test by the mice 1 hour after unique oral administration of EE, and (g and h) percentage of the mice with loose stools after unique oral administration of EE. *n* = 10 (5 females + 5 males). ⁣^∗^*p* < 0.05 and ⁣^∗∗^*p* < 0.01 show a significant difference compared to the naïve group (one-way ANOVA with Dunnett's multiple comparisons posttest). EE: ethanol extract.

To examine the safety of *A. sieberiana*, the acute toxicity test was performed. The results of the acute toxicity test are presented in Figure 10. Irrespective of sex, EE did not induce any significant change in body mass (Figures 10(a), 10(b), 10(c), and 10(d)). The locomotor activity of females was not affected by the plant extract treatment, while males receiving EE at doses of 1800 and 5400 mg/kg showed a significant reduction in the total distance traveled (Figures 10(e) and 10(f)). At these doses, irrespective of sex, 20% and 40% of mice had loose stools (Figures 10(g) and 10(h)). However, no mortality or other signs of toxicity were recorded in mice treated with EE during the 14 days of observation.

## 4. Discussion

This study aimed to evaluate the therapeutic effects of AE and EE of the stem bark of *A. sieberiana* on a mouse model of pain–depression induced by reserpine. Our main findings are the reversal of reserpine-induced nociception (by mitigating tactile and cold allodynia) and depression by enhancing the mobility of animals. Both extracts modulate a number of pain–depression–related biochemical parameters, including neurotransmitters, cytokines, and oxidative and nitrosative pathways. In addition, EE was the most effective extract and did not show significant adverse effects.

The reserpine-induced pain–depression dyad has been developed as a suitable animal model that mimics the clinical condition, and it is widely used in drug discovery experiments [13, 59, 60]. Reliably, we showed in this work that three consecutive days of injection of reserpine in mice at 1 mg/kg/day resulted in the establishment of a pain–depression dyad. In fact, reserpine-treated animals exhibited an increased response to acetone application (cold allodynia) and von Frey hairs (mechanical allodynia). These findings are in accordance with previous results [61]. Reserpine-induced allodynia results from an ASIC3-increased response in the dorsal root ganglion in C-nociceptors that is intensified by spinal microglia activation [60, 62]. Further pain amplification comes from the dysfunction of the descending inhibitory system of pain. *A. sieberiana* treatment reversed the increase in nociception induced by reserpine administration in mice. This finding suggests that *A. sieberiana* extracts may have inhibitory effects on C-fibers and microglial activation or regulate the pain descending inhibitory system. One of the interesting aspects of this reserpine model is central sensitization at the level of Laminae I-II of the spinal cord and peripheral modifications [60]. Considering this, it can be thought that *A. sieberiana* extract could reduce central sensitization or inhibit peripheral nociceptors. Tactile and cold allodynia are due to modifications such as a loss of integrity of Aδ and overexpression and hyperactivation of several receptors, such as TRPs [63, 64]. The antiallodynic effect of AE and EE suggests that the analgesic effects of these plant extracts could be mediated through the inhibition of TRP channels, including TRPA1 and/or TRPM8, which are specific to cold allodynia.

It is well established that increased nociception is coupled with depression. Several tests are known to evaluate depression-like behavior in animals, including FST, TST [65, 66], and the pole test [67] that were used in the present study. Herein, reserpine administration induced a reduction in motor activity in animals in the pole test and increased immobility time in the FST and TST. Treatment with *A. sieberiana* extracts dose- and time-dependently increased motor coordination in the pole test and motility function in the TST. These results suggest the antidepressant properties of *A. sieberiana*. It is worth noting that a single administration was less active, suggesting the effect of plant extracts through time-demanding processes. It was, however, surprising that *A. sieberiana* extracts had no effect on the immobility time in the FST, while a strong negative correlation has been demonstrated between PWT and FST immobility time in reserpinized animals [60]. However, in the course of the present study, the FST was performed 5 h after administration. This could be a limit in assessing the activity of these plant extracts, as their pharmacokinetics are unknown.

Neurotransmitters are vital biochemical molecules that control behavioral and physiological functions in the central and peripheral nervous systems [68]. In particular, the metabolic disturbance of monoamine neurotransmitters, especially 5-HT, is closely linked with neuropsychiatric disorder development [69], such as depression. Concordantly, the depression-like behavior developed by reserpine-treated animals was associated with a significant decline in brain total catecholamine and serotonin concentrations but not dopamine. Intraperitoneal administration of L-tryptophan and oral administration of *A. sieberiana* extracts reversed the alteration in catecholamine concentration and even increased the dopamine content in the brain. This result could therefore justify the reduction in the time of immobility observed during the TST and the improvement in motility in the pole test. However, it is worth noting that the plant extracts were unable to significantly reverse the drop in serotonin concentration induced by reserpine. This finding supports the recent hypothesis that serotonin is not the main mediator of depression [70, 71]; given that *A. sieberiana* extracts were unable to restore the brain serotonin content despite their antidepressant effect. Nevertheless, these data suggest that the analgesic and antidepressant effects of *A. sieberiana* might be mediated via the modulation of cerebral biogenic amines.

Reserpine, via a blockade of the vesicular monoamine transporter for neuronal transmission or storage, also promotes dopamine autoxidation and oxidative catabolism by monoamine oxidase, which leads to the formation of dopamine quinones and hydrogen peroxide, resulting in oxidative stress and lipid peroxidation [72]. The accumulation of ROS and reactive quinone leads to cell damage and the activation of proinflammatory signals [73]. The activation of microglia by TNF-α and IL-1β amplifies proinflammatory signaling, resulting in nitrite oxide and peroxynitrite accumulation, as well as the catabolism of tryptophan by indoleamine dioxygenase (IDO), which leads to neurotoxic catabolites, such as kynurenine, to trigger inflammation [74]. These physiological and biochemical processes implicate inflammation and oxidative stress in the sustainability of the pain–depression dyad both in the animal model used herein and in clinical conditions and further justify the evaluation of these parameters in the present work.

MDA is an indicator of lipid peroxidation [75]. In depression, MDA levels increase in brain tissue [76], while the levels of the antioxidant enzymes GSH, CAT, and SOD are reduced [19, 25, 77]. In reserpine-treated mice, we showed higher levels of MDA and decreased activity of SOD in the spinal cord and decreased CAT and GSH activity in the brains of these mice. Remarkably, *A. sieberiana* extracts reduced MDA levels and increased the activity of antioxidant enzymes, suggesting the antioxidant potential of this plant. The concentration of nitrite oxide also increased in the brain and spinal cord of mice in response to reserpine administration, and this increase was reversed by treatment with *A. sieberiana* extracts. An increase in NO concentration is related not only to nitrosative stress but also to inflammatory processes. In fact, NO overproduction as well as the recruitment of glial cells leads to excessive production of proinflammatory cytokines (TNF-α, IL-1β, and IL-6), which are involved in not only the initiation but also the persistence of pathologic pain by directly reducing the nociceptor stimulation threshold [78]. Several studies have also shown that depression and anxiety are associated with higher levels of inflammation [79, 80]. [60] showed that repeated injection of reserpine leads to an increase in proinflammatory cytokines. This result is concordant with the findings of the present study, which further demonstrates that repeated administration of *A. sieberiana* extracts dose-dependently reduced serum IL-1β and TNF-α levels, suggesting an anti-inflammatory effect of this plant. AE and EE failed to reduce brain cytokines impaired by reserpine. These surprising results may infer that the increased cytokines found in the bloodstream are not produced in the CNS.

Oxidative stress in depression is also a major cause of dysfunction of neurotransmitter modulation in the brain [81]. A significant increase in AChE activity in the brains of mice with depression phenotypes and a positive correlation between AChE and O_2_^•−^ levels associated with depressive behaviors have been demonstrated [31]. This finding therefore suggests that oxidative stress may induce overactivation of AChE, leading to depression-related behaviors. Furthermore, a direct activation of cholinergic receptors or extending the action of endogenous acetylcholine via pharmacological blockade of AChEreduces pain in rodents as well as humans [29]. Consistently, we showed that reserpine-induced allodynia, oxidative stress parameters, and AChE activity in mice. Interestingly, *A. sieberiana,* irrespective of the dose and the type of extract, significantly reduced the activity of AChE. This reduction in AChE activity may at least partially justify the analgesic potential of the plant extracts and might be related to their antioxidant properties.

From a global consideration of the results, it appears that female mice responded less to the analgesic effect of *A. sieberiana* extracts. These results are consistent with several studies that showed sex-differential responses to analgesics [82–84]. Testosterone has consistently shown its protective role against pain in men, leading to a lower prevalence of chronic pain in men than in women [85, 86]. The marked analgesic effect of the extracts in males compared to females may be due to the high testosterone level in males. Pain is closely linked to the overexpression of proinflammatory cytokines, and studies have shown that this overexpression depends on hormonal variation in female and male animals. However, the literature reports that both testosterone and estrogens limit the production of proinflammatory cytokines such as TNF-α [87]. Concordantly, our results showed no sex difference in TNF-α levels in the brain and serum. Nevertheless, a reduced level of IL-1β was observed in the female's brain as compared to the male. These results suggest that sex-related variation of cytokines depends on the type of cytokine. Besides, as the hormonal status of females was not assessed in the present study, it is difficult to draw a powerful conclusion on this point.

In addition, gender analysis showed that the antidepressant effect of *A. sieberiana* extracts was marked in both females and males, but females tended to be more sensitive to the treatment. Although there is a clear evidence of a sex effect both on the prevalence of depression and the response to treatment, the effect of sex hormones on depression-related neurotransmitters seems to be unidirectional. In fact, both estrogens and androgens modulate neurotransmitters in the brain, including serotonergic, noradrenergic, GABAergic, dopaminergic, and glutamatergic systems, which will therefore affect cognition, attention, motor activity, fine motor skills, and mood regulation in animals [88–90]. Thus, a drop in estrogen and testosterone can lead to increased levels of sadness, anxiety, and irritability. Therefore, sex hormones are unlikely to explain the sex difference in depression. Nevertheless, testosterone affects the noradrenergic but not the serotonergic system [71, 91].

Taken together, this study showed that *A. sieberiana* reversed the pain–depression dyad induced in a mouse model by reserpine injection and that EE had a greater effect. The greater effect of EE as compared to AE, might be due to differences in chemical composition, either quantitatively or qualitatively. This will be explored in the future; however, for this publication, we only focused on the constituents of the more active fraction. It was essential to perform a toxicological evaluation of the ethanol extract for its safety in potential therapeutic applications. The study showed that the single administration of high doses of *A. sieberiana* up to a dose of 5400 mg/kg did not cause mortality in female and male mice, inferring that the LD_50_ would be greater than 5400 mg/kg. Thus, according to the Globally Harmonized Classification System (GHS), the ethanol extract of *A. sieberiana* can be classified as a relatively nontoxic substance [92]. However, loosed stools were observed in animals treated with higher doses of EE, suggesting that this extract might induce diarrhea.

The phytochemical profiling of the ethanol extract showed the presence of 5 compounds including piperine, aurantiamide acetate, and asperphenamate along with the likely presence of a number of flavonoids that might account for the observed pharmacological activities. Indeed, analgesic, anti-inflammatory, and antidepressant effects of piperine have been demonstrated in various animal models [94–96]. Apigenin also demonstrated anti-inflammatory and antidepressant activity [97], while aurantiamide acetate showed analgesic and anti-inflammatory properties [98, 99]. Moreover, Olayinka et al. [100] recently demonstrated that apigenin mitigates depression-like behavior by modulating monoamine oxidase enzyme, a mechanism close to what is proposed for *A. sieberiana* extracts.

## 5. Conclusion

AE and EE of *A. sieberiana* trunk bark are effective against the pain and depression dyad induced by reserpine in mice. The sex-related activity indicates that males respond better to the analgesic treatment, while females seem to be more prone to the antidepressant effect. The analgesic and antidepressant activities of the plant extracts are probably related to their anti-inflammatory, antioxidant, and catecholamine modulatory effects in the brain, spinal cord or serum. EE is the most effective extract and can be classified as a relatively nontoxic substance. Its pharmacological activities may be ascribed to the presence of piperine, aurantiamide acetate, and apigenin. This study shows for the first time the analgesic and antidepressant effects of *A. sieberiana* and further evokes the mechanism of action. Further studies are needed for long-term toxicity, and to test the pharmacological effect on different models and animal species [96].

## Figures and Tables

**Table 1:** Effect of aqueous and ethanol extracts of *Acacia sieberiana* on oxidative stress parameters and nitrite oxide in brain and spinal cord tissues.

	Treatment	MDA (μmol/g tissue)	SOD (U/mg protein)	CAT (H_2_O_2_/mg protein/min)	GSH (μmol/g tissue)	NO (μmol/g tissue)
Brain	Naïve	10.36 ± 0.24	2.70 ± 0.29	49.08 ± 2.01	147.97 ± 14.82	7.43 ± 0.76
	Control	9.96 ± 0.36	3.56 ± 0.23	48.77 ± 3.39	144.70 ± 11.22	16.20 ± 3.40⁣^∗∗∗^
	L-trypt 100 mg/kg	11.01 ± 0.61	3.74 ± 0.46	39.20 ± 4.32	121.58 ± 17.70	8.67 ± 1.20^$$^
	AE 25 mg/kg	9.36 ± 0.51	2.87 ± 0.17	53.84 ± 2.63	169.70 ± 25.55	6.30 ± 1.17^$$$^
	AE 50 mg/kg	9.44 ± 0.22	3.25 ± 0.23	51.99 ± 5.11	250.04 ± 30.57^$^	4.73 ± 0.62^$$$^
	AE 100 mg/kg	10.04 ± 0.52	3.32 ± 0.27	61.72 ± 1.16^$,##^	234.07 ± 46.24	2.40 ± 0.78^$$$,#^
	EE 25 mg/kg	7.18 ± 0.35^$$,#^	2.40 ± 0.25	56 ± 3.36	360.34 ± 59.08^$$,#^	1.40 ± 0.32^$$$,###^
	EE 50 mg/kg	9.48 ± 0.37	2.89 ± 0.38	50.93 ± 4.39	600.01 ± 47.68^$$$,###^	3.02 ± 0.83^$$$^
	EE 100 mg/kg	8.65 ± 0.96	3.00 ± 0.28	47.58 ± 3.21	518.03 ± 71.08^$$$,#^	4.97 ± 0.45^$$$^

Spinal cord	Naïve	6.32 ± 0.49	0.74 ± 0.09		179.89 ± 30.60	250.43 ± 34.18
	Control	7.48 ± 0.48	0.14 ± 0.04⁣^∗∗∗^		121.79 ± 18.55	507.22 ± 31.91⁣^∗∗∗^
	L-trypt 100 mg/kg	6.64 ± 0.42	0.19 ± 0.04		145.75 ± 17.38	541.37 ± 32.28
	AE 25 mg/kg	8.43 ± 0.44	0.24 ± 0.05		148.45 ± 20.37	482.75 ± 48.38
	AE 50 mg/kg	8.82 ± 0.30	0.42 ± 0.08^$^		206.96 ± 38.38	302.77 ± 49.53^$$^
	AE 100 mg/kg	7.91 ± 1.15	0.24 ± 0.06		173.74 ± 34.06	101.61 ± 16.98^$$$^
	EE 25 mg/kg	7.48 ± 0.77	0.66 ± 0.11^$$$,##^		214.93 ± 22.35	90.04 ± 10.30^$$$,###^
	EE 50 mg/kg	4.46 ± 0.44^$$,###^	0.48 ± 0.04^$^		288.23 ± 34.09^$$$^	101.32 ± 9.99^$$$,#^
	EE 100 mg/kg	7.97 ± 0.88	0.53 ± 0.10^$^		219.49 ± 36.48	101.54 ± 14.08^$$$^

*Note: n* = 10 (5 females + 5 males). GSH, glutathione.

Abbreviations: AE, aqueous extract; CAT, catalase; EE, ethanol extract; L-trypt, L-tryptophan; MDA, malondialdehyde; NO, nitrite oxide; RES + DW, reserpine + distilled water; SOD, superoxide dismutase.

⁣^∗^*p* < 0.05, ⁣^∗∗^*p* < 0.01, and ⁣^∗∗∗^*p* < 0.001 show a significant difference compared to the naïve group.

^$^ *p* < 0.05, ^$$^*p* < 0.01, and ^$$$^*p* < 0.001 show a significant difference from control.

^#^ *p* < 0.05, ^##^*p* < 0.01, and ^###^*p* < 0.001 show a significant difference between the two extracts at the same dose.

## Data Availability

The data that support the findings of this study are available from the corresponding author upon reasonable request.

## Supporting Information

The attached supporting file contains detailed methods and analysis of the ethanol extract using molecular networking.
