# Supplementary material for: Aqueous and Ethanol Extracts of Acacia sieberiana (Fabaceae) Stem Bark Reverse the Pain–Depression Dyad in Mice Through Modulation of Catecholamines, Proinflammatory Cytokines, and Oxidative Stress
Source: Adv Pharmacol Pharm Sci. 2025 Feb 28;2025:1244498. doi: 10.1155/adpp/1244498 (PMC11991813; doi:10.1155/adpp/1244498)

Supplemental Information

**Aqueous and ethanol extracts of Acacia sieberiana (Fabaceae) stem bark reverse the pain-depression dyad in mice through modulation of catecholamines, pro-inflammatory cytokines, and oxidative stress**

Sorelle Ngassam Mbankou, Aliance Romain Fokoua, Cedric Wamba Koho, Roger Hermann Sadie Foguieng, Sahar Mofidi Tabatabaei, Pamela Arielle Nono Nankam, Kevin Joseph Tidgewell, Télesphore Benoît Nguelefack

Table of Contents:

| Detailed Methods for LC-MS/MS Molecular Networking Analysis | pg 3 |
| --- | --- |
| S1: Molecular Networking analysis of A. sieberiana ethanolic extract (EE) using LC method A and MS method 1 | pg 4 |
| S2: Extracted ion chromatogram and MS/MS spectral comparison (experimental vs. library) for piperine | pg 4 |
| S3: Extracted ion chromatogram and MS/MS spectral comparison (experimental vs. library) for piperolein B | pg 5 |
| S4: Extracted ion chromatogram and MS/MS spectral comparison (experimental vs. library) for aurantiamide acetate | pg 5 |
| S5: Molecular Networking analysis of EE using LC method B and MS method 2 | pg 6 |
| S6: Extracted ion chromatogram and MS/MS spectral comparison (experimental vs. library) for asperphanamate | pg 6 |
| S7: Molecular Networking analysis of EE using LC method B and MS method 1 | pg 7 |
| S8: Extracted ion chromatogram and MS/MS spectral comparison (experimental vs. library) for beauvericin | pg 8 |
| S9: Molecular Networking analysis of EE using LC method C and MS method 1 | pg 9 |
| S10: Extracted ion chromatogram and MS/MS spectral comparison (experimental vs. library) for 5, 6, 2’- trimethoxyflavone | pg 9 |
| S11: Molecular Networking analysis of EE using LC method D and MS method 2 | pg 10 |
| S12: Extracted ion chromatogram and MS/MS spectral comparison (experimental vs. library) for apigenin | pg 10 |
| S13: Extracted ion chromatogram and MS/MS spectral comparison (experimental vs. library) for hydnocarpin | pg 11 |
| S14: Molecular Networking analysis of EE using LC method D and MS method 2 in negative mode | pg 11 |
| S15: Extracted ion chromatogram and MS/MS spectral comparison (experimental vs. library) for 6, 3’-dihydroxyflavone | pg 12 |

**Detailed Methods for LC-MS/MS Molecular Networking Analysis**

A 4μm Polar-RP Phenomenex C18 column (150 × 4.6 mm) was maintained at room temperature and eluted at a flow rate of 0.5 ml/min with water + 0.1% formic acid (solvent A) and acetonitrile (solvent B) using the following gradients: Method A: 0–5 min, 60% B; 5–22 min, to 85% B, 22–25 min, 85% B; 25–27 min, to 100% B; 27–32 min, 100% B. Method B: 0–5 min, 70% B; 5–22 min, to 85% B, 22–25 min, 85% B; 25–27 min, to 100% B; 27–32 min, 100% B. Method C: 0–5 min, 40% B; 5–22 min, to 70% B, 22–25 min, 70% B; 25–27 min, to 100% B; 27–32 min, 100% B. Method D: 0–5 min, 20% B; 5–22 min, to 70% B, 22–25 min, 70% B; 25–27 min, to 100% B; 27–32 min, 100% B.

MS/MS spectra were acquired using an IonSpray voltage floating 5500 V, and collision energy of 3500 V. The source temperature was set at 500°C, with an ion source gas 1 and 2 flow of 50 L/h and 55 L/h, respectively. MS/MS survey scans were set up as dependent acquisition where the 20 most intense ions detected in the MS spectrum were selected as MS/MS precursors. Typically, the threshold for MS/MS targeting was set to 100 with targeted ions subsequently excluded from targeting for 15 s for one method (MS method 1) and never excluded for the second method (MS method 2). The data were clustered with the GNPS2 website with a parent mass tolerance of 2 Da and a MS/MS fragment ion tolerance of 0.5 Da to create molecular networks where each node is a consensus MS/MS spectrum and edges between the nodes indicate the degree of similarity between consensus spectra. Networks were created in which edges were filtered to have a cosine score above 0.7 and more than six matched peaks.

**S1**: Molecular Networking analysis of *A. sieberiana* ethanolic extract (EE) using LC method A and MS method 1


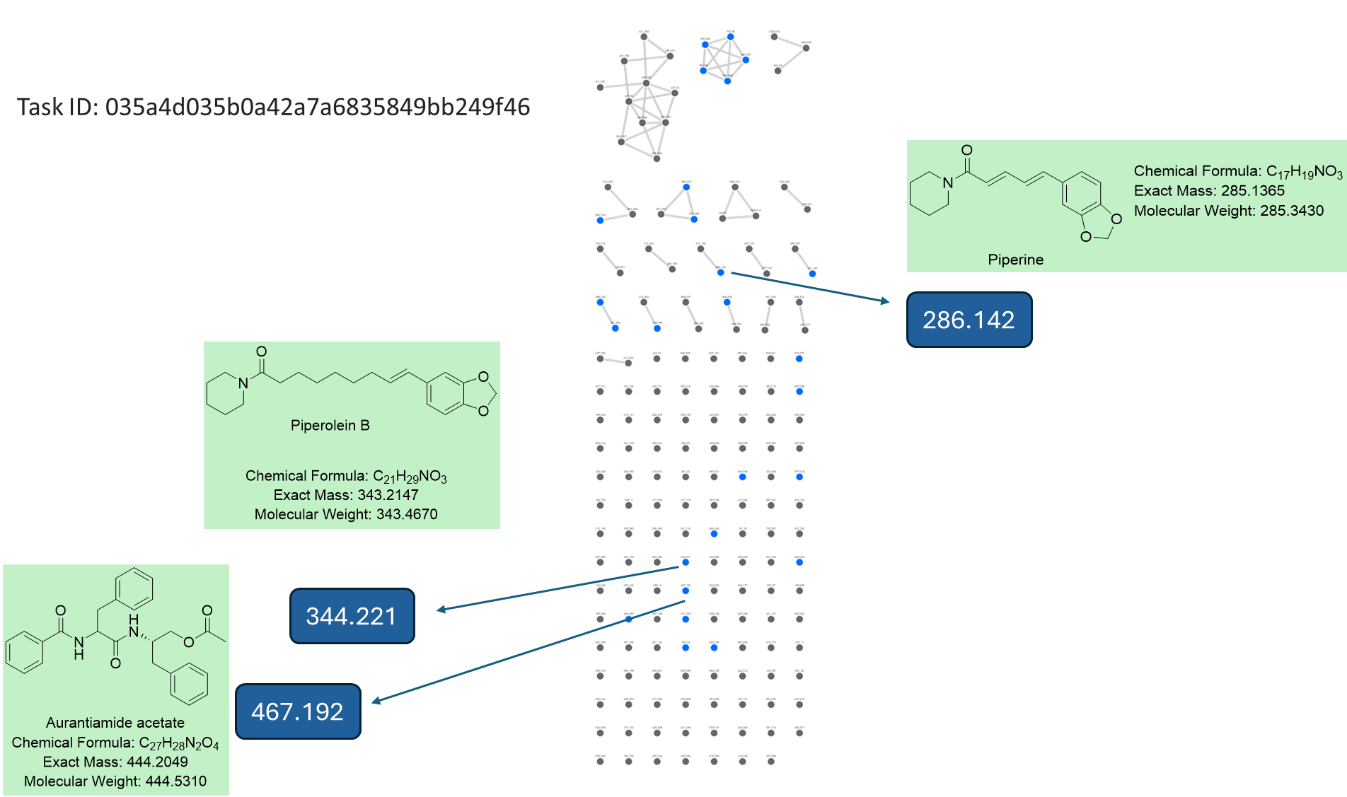


**
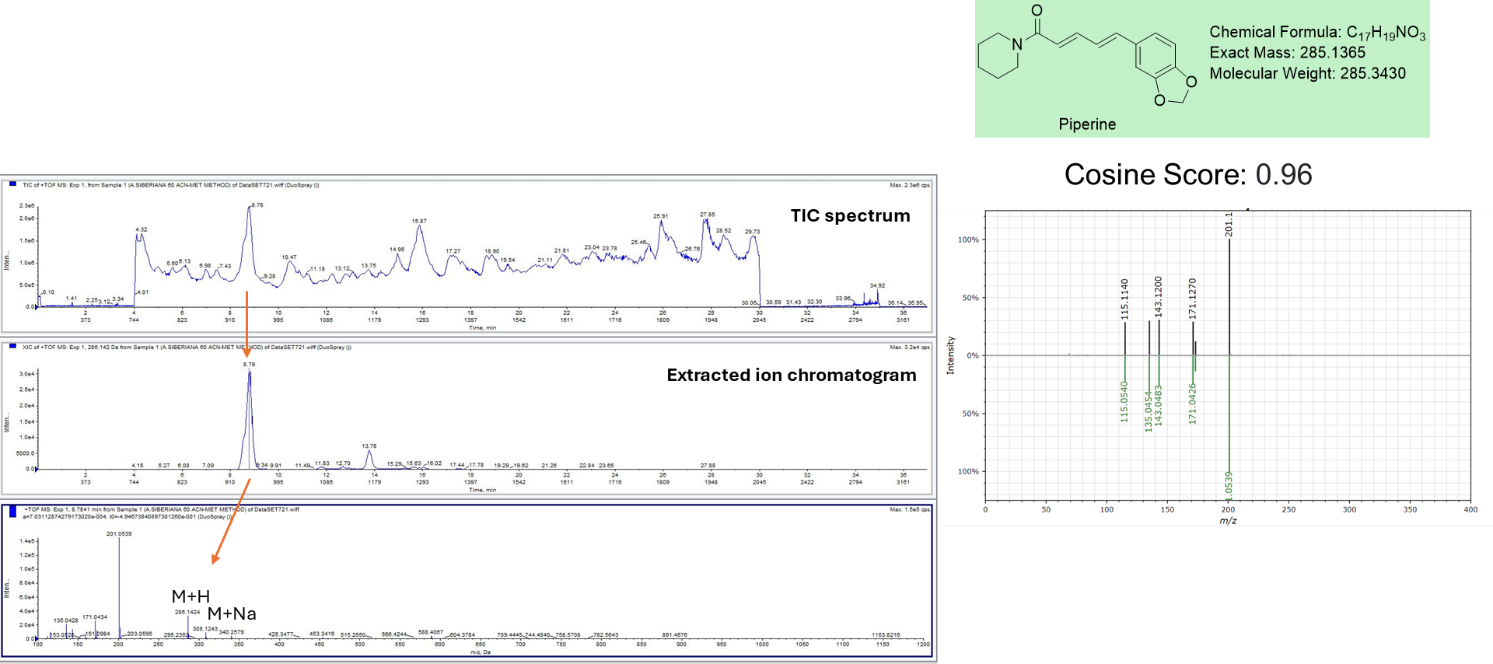
S2**: Extracted ion chromatogram and MS/MS spectral comparison (experimental vs. library) for piperine

**S3**: Extracted ion chromatogram and MS/MS spectral comparison (experimental vs. library) for piperolein B


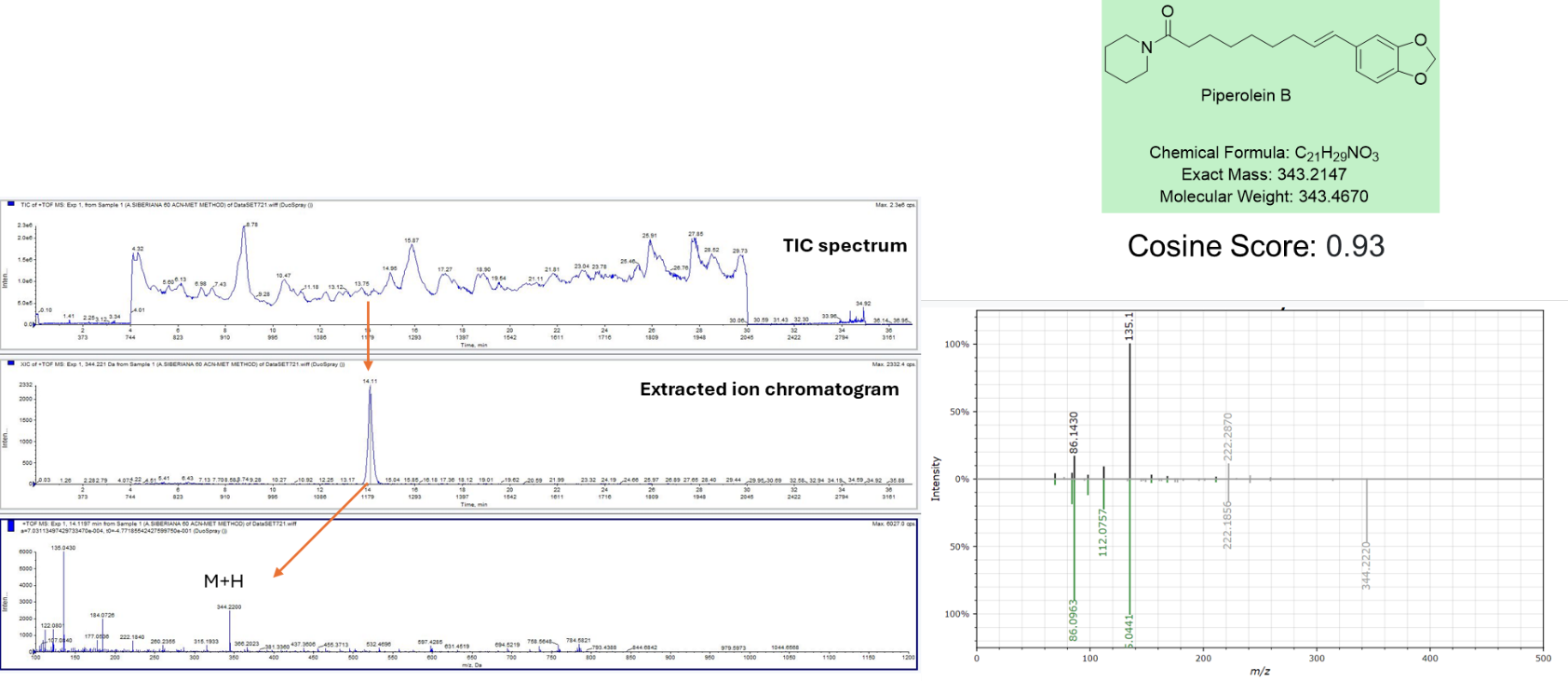


**S4:** Extracted ion chromatogram and MS/MS spectral comparison (experimental vs. library) for aurantiamide acetate


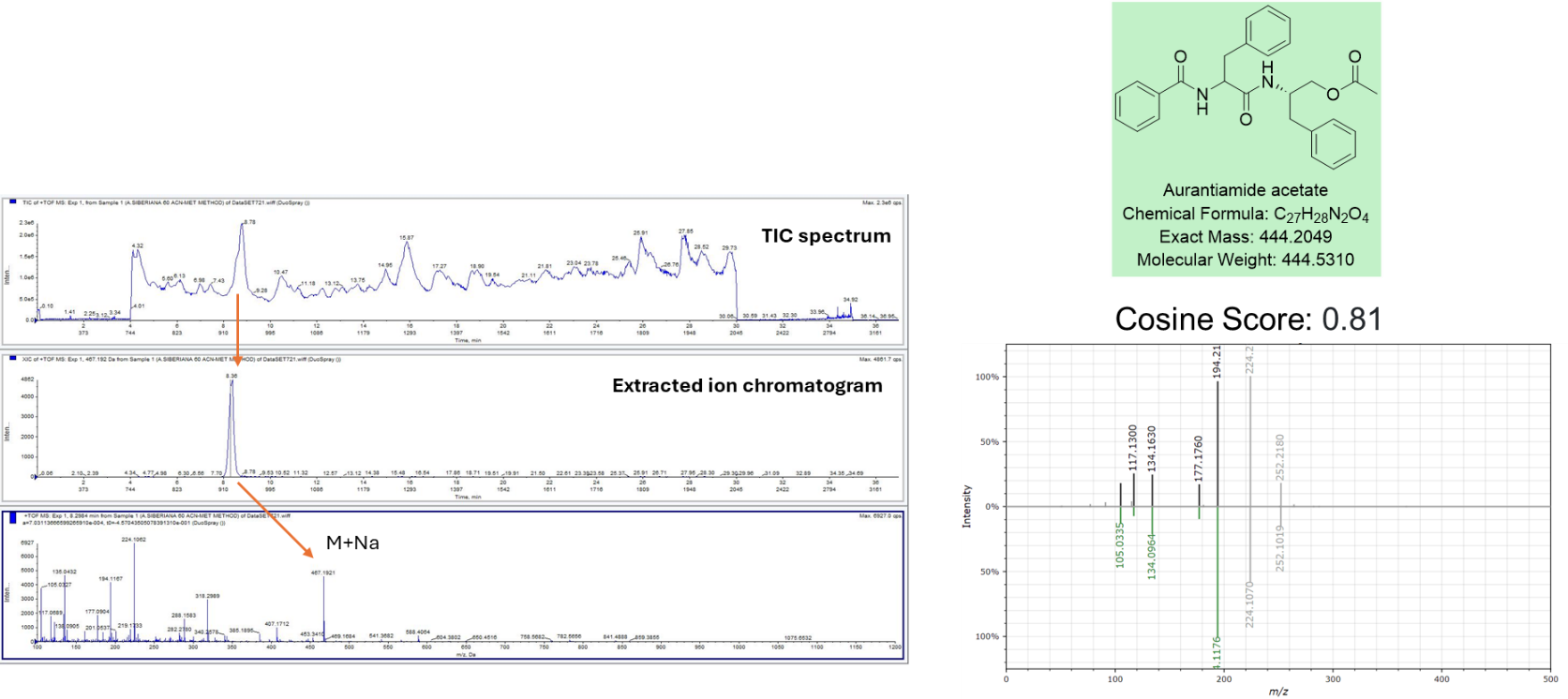


**S5**: Molecular Networking analysis of EE using LC method B and MS method 2


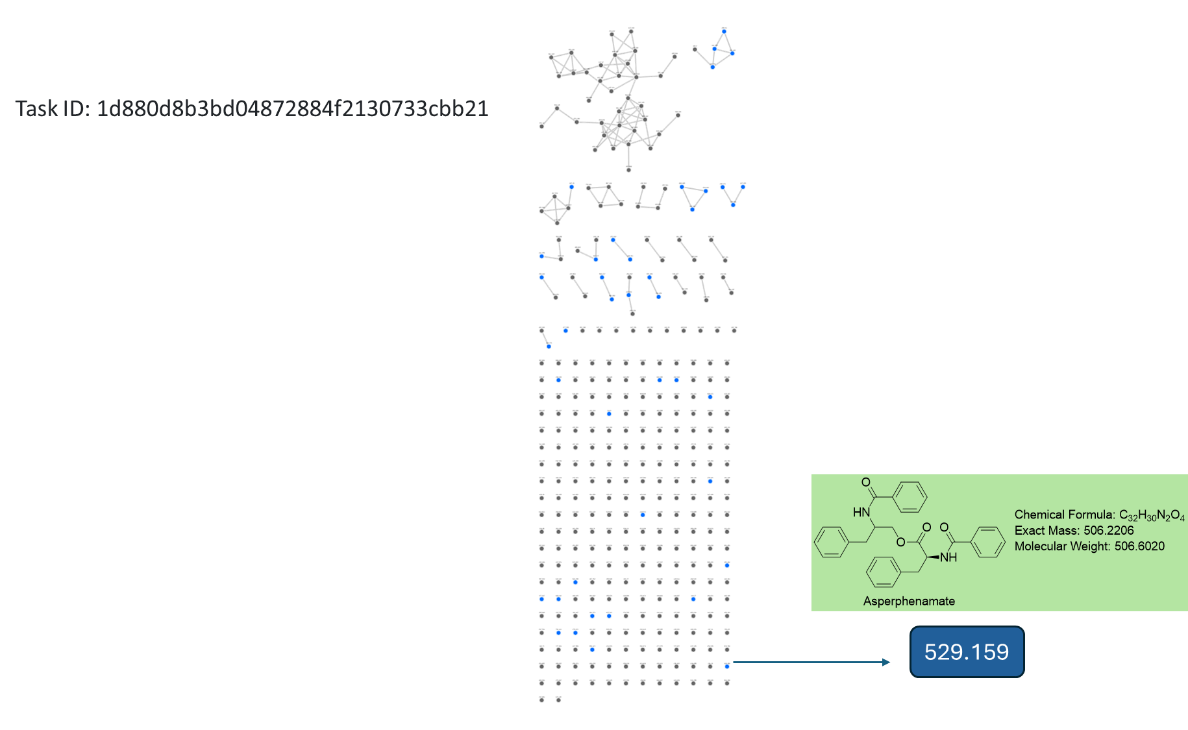


**S6**: Extracted ion chromatogram and MS/MS spectral comparison (experimental vs. library) for asperphanamate


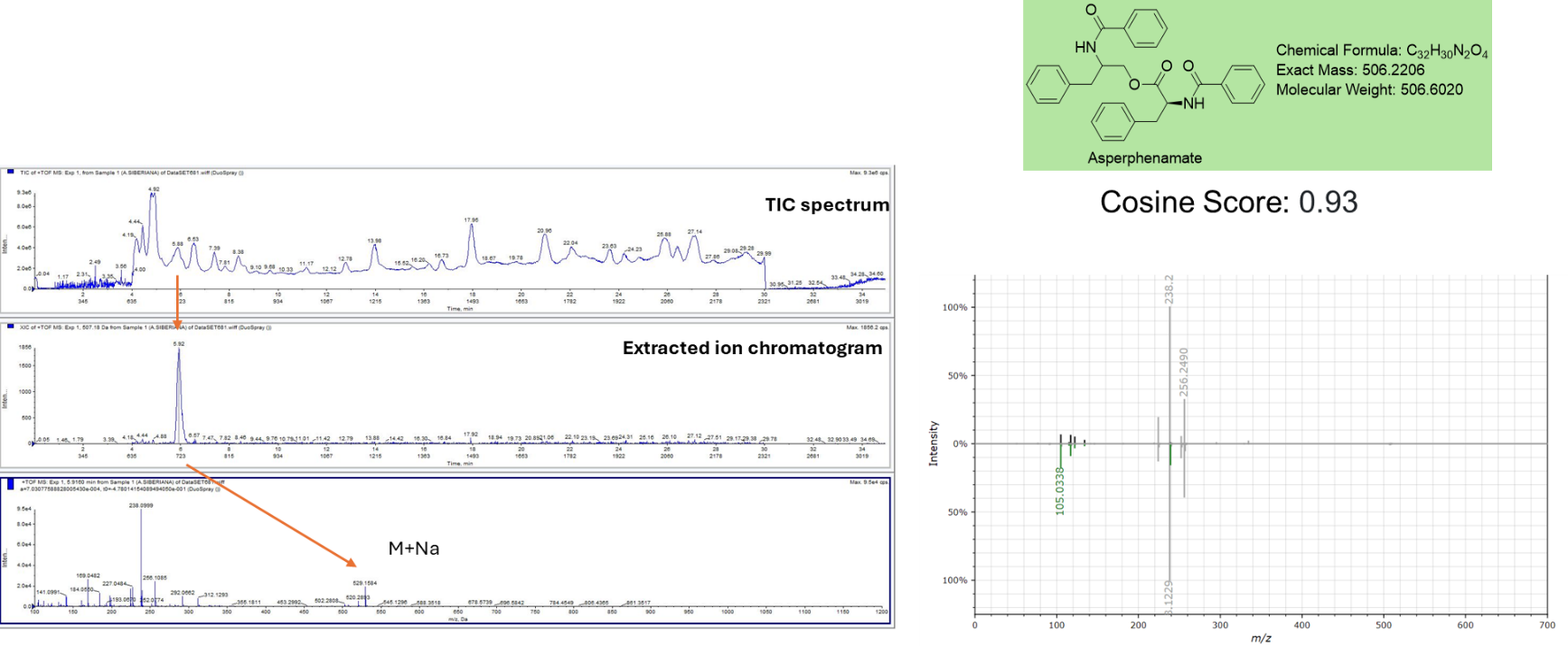


**S7**: Molecular Networking analysis of EE using LC method B and MS method 1


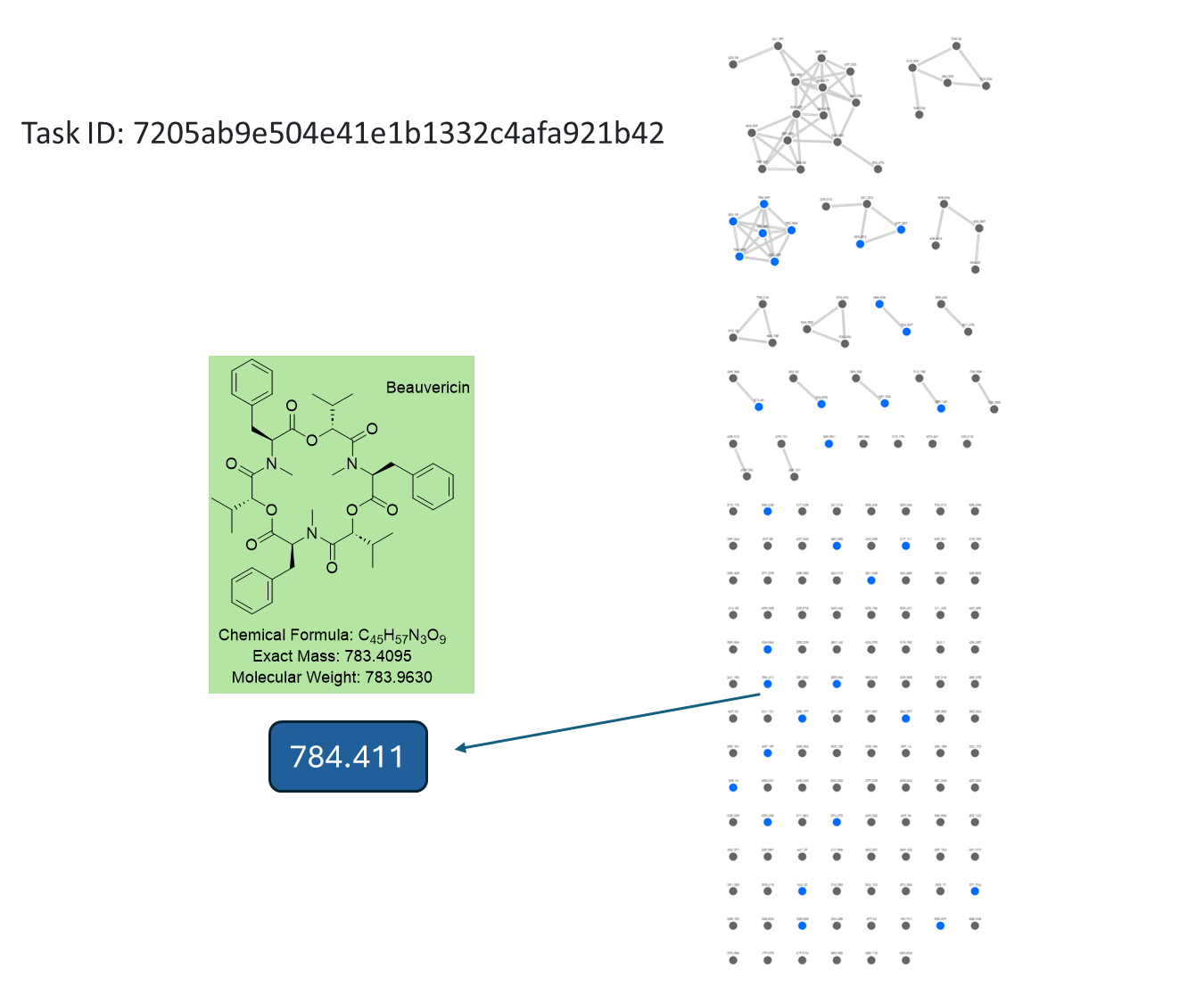


**S8**: Extracted ion chromatogram and MS/MS spectral comparison (experimental vs. library) for beauvericin


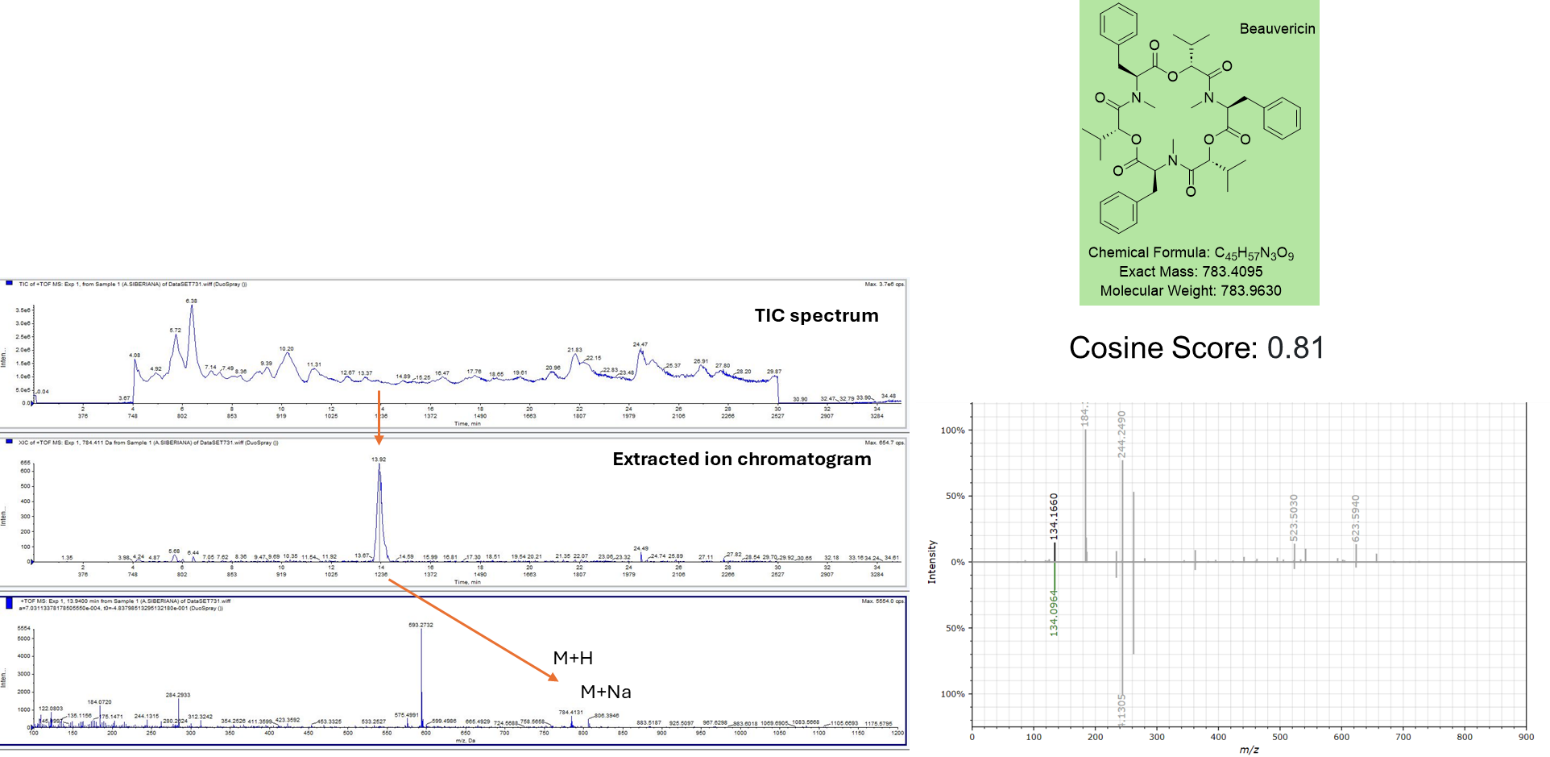


**S9**: Molecular Networking analysis of EE using LC method C and MS method 1


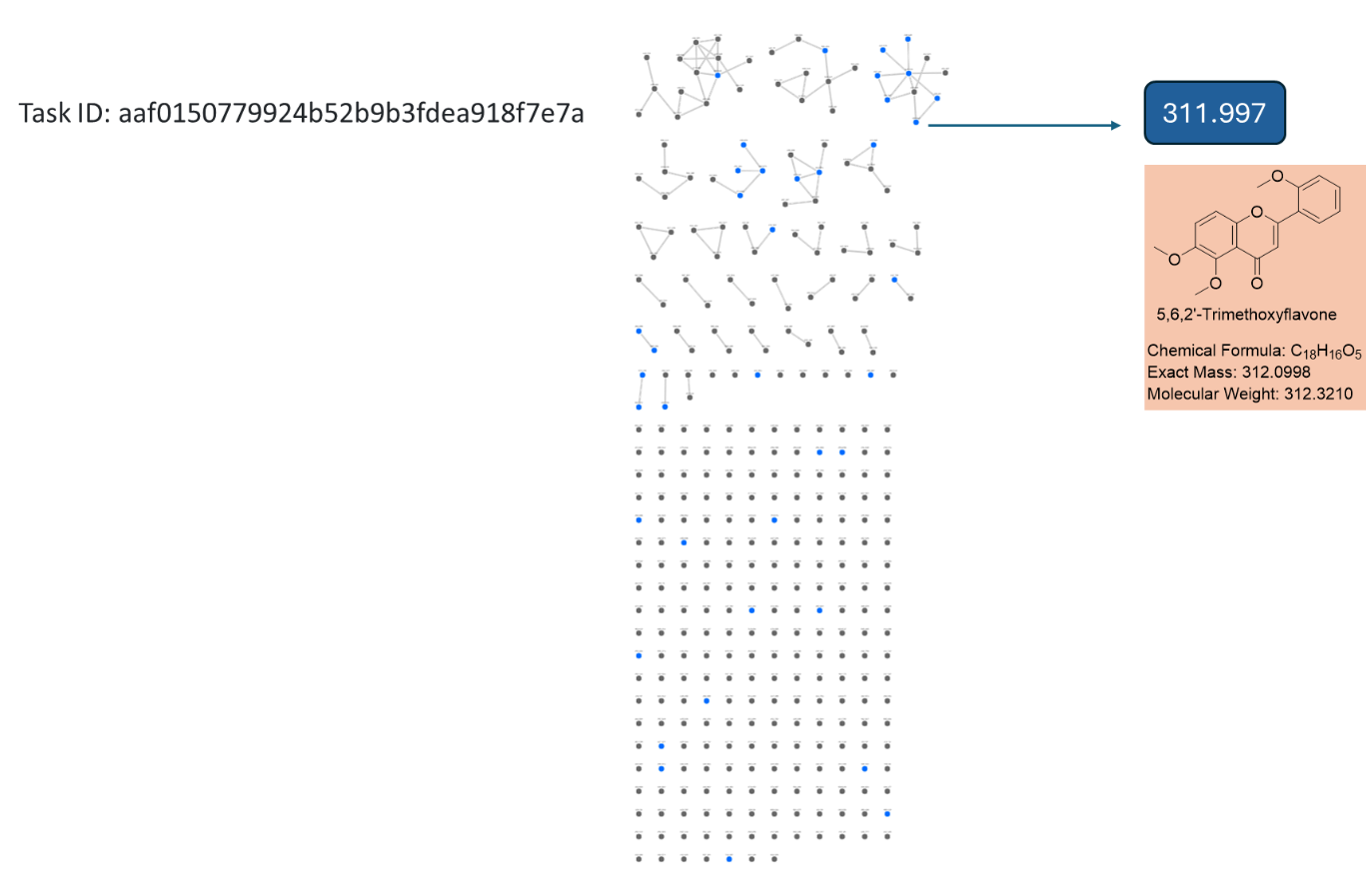


**
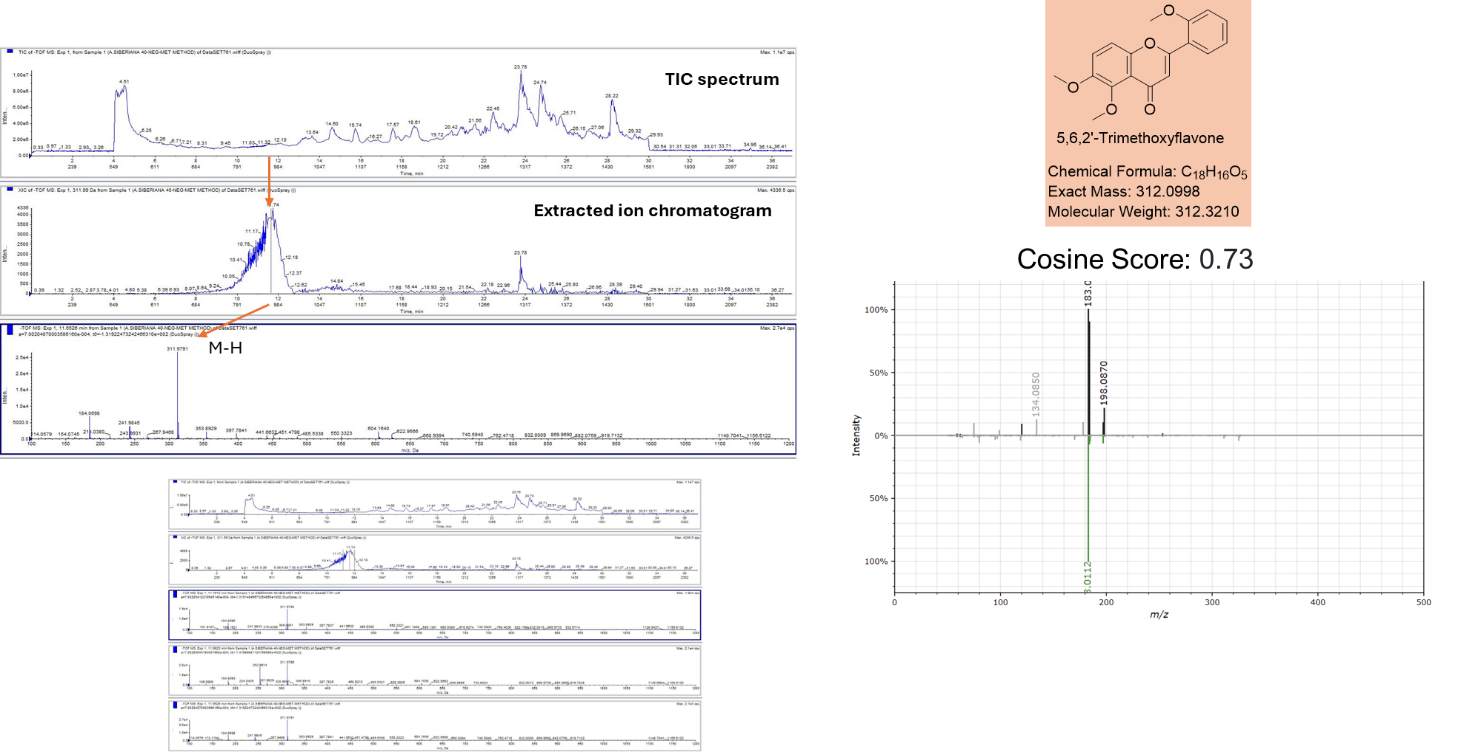
S10**: Extracted ion chromatogram and MS/MS spectral comparison (experimental vs. library) for 5, 6, 2’- trimethoxyflavone

**S11**: Molecular Networking analysis of EE using LC method D and MS method 2


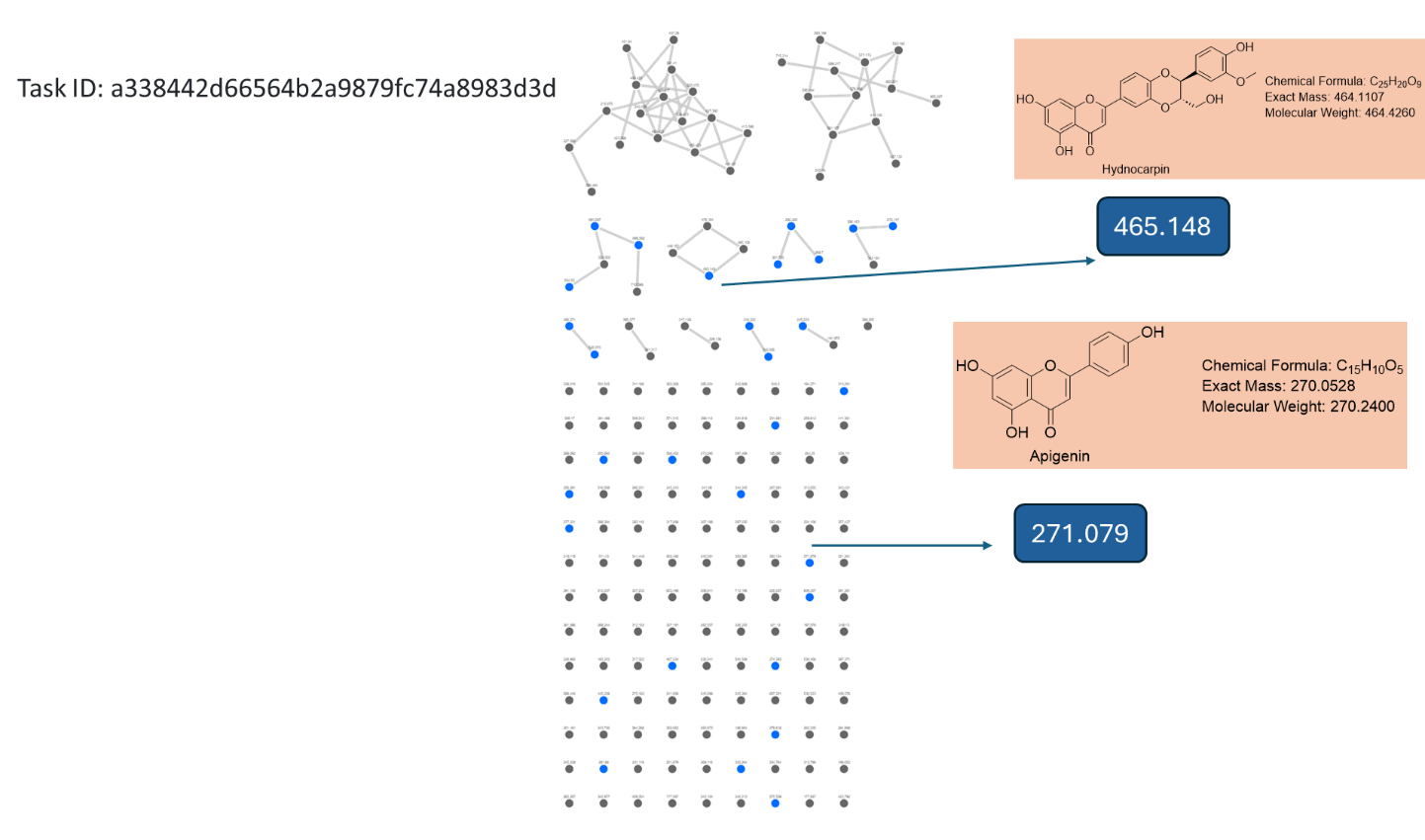


**S12**: Extracted ion chromatogram and MS/MS spectral comparison (experimental vs. library) for apigenin


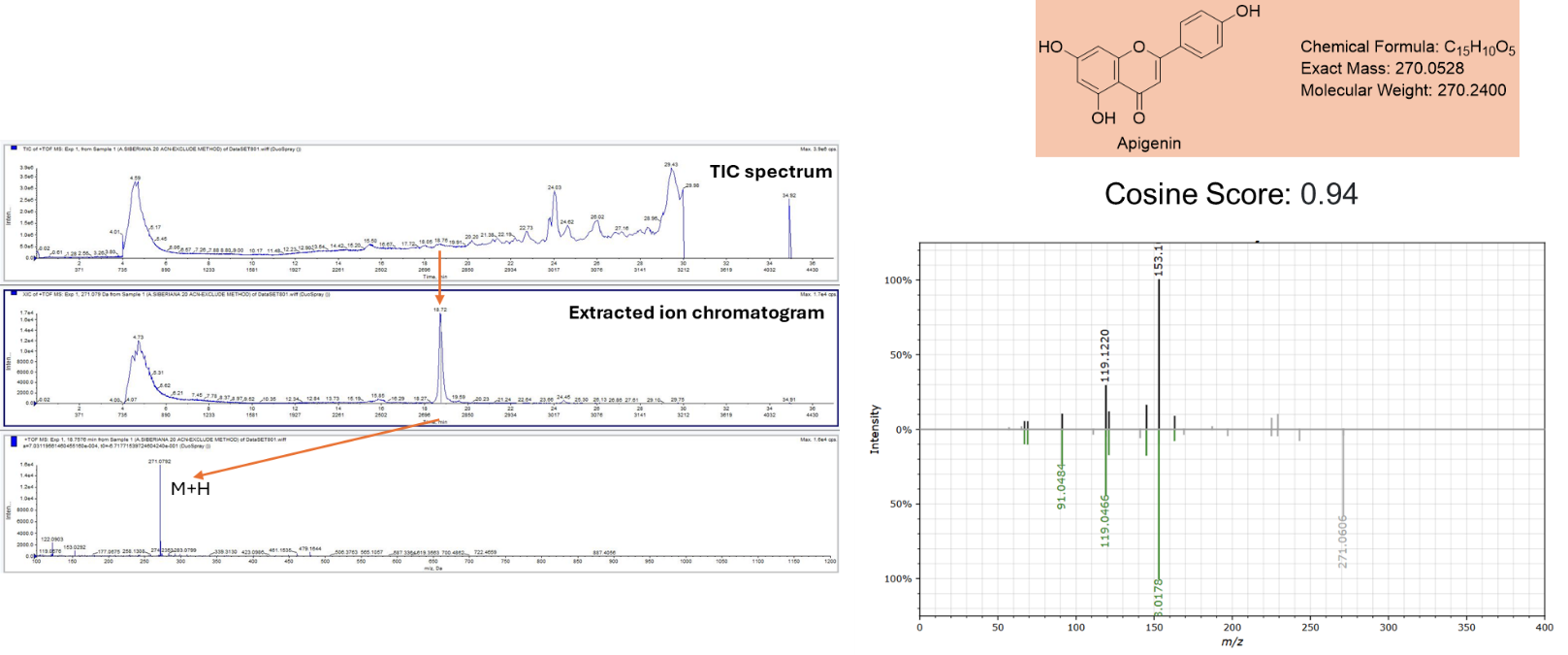


**S13**: Extracted ion chromatogram and MS/MS spectral comparison (experimental vs. library) for hydnocarpin


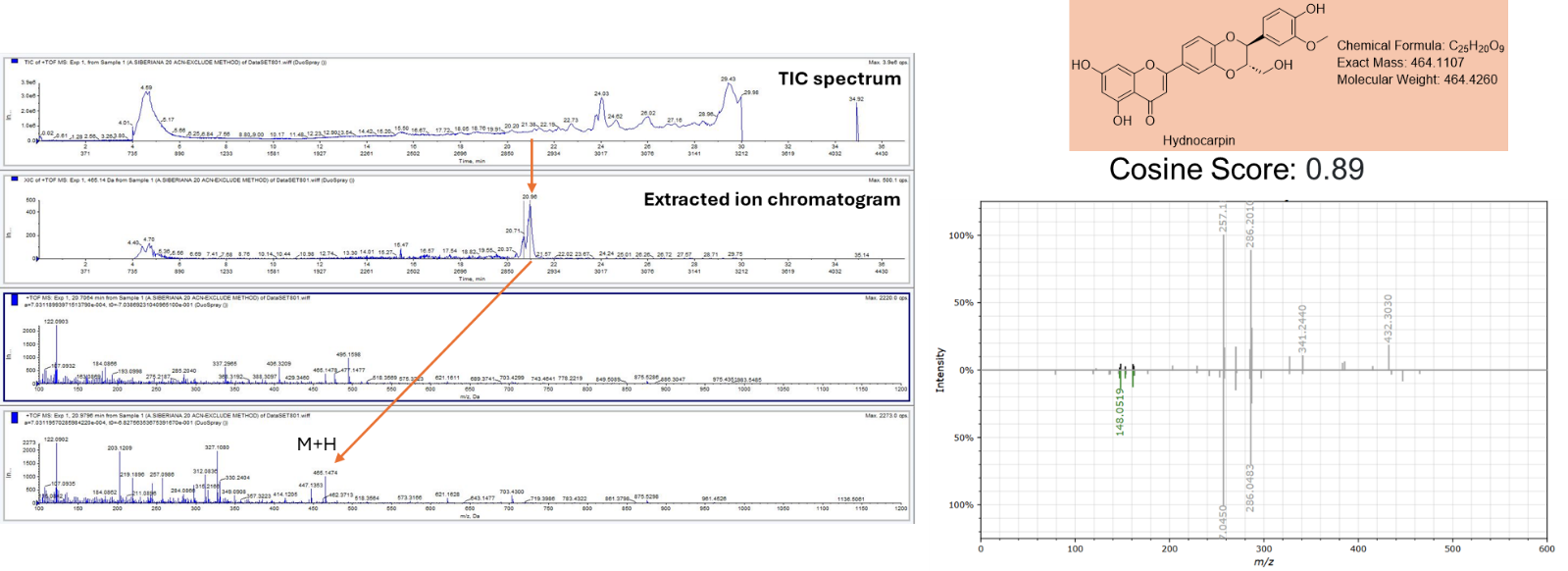


**S14**: Molecular Networking analysis of EE using LC method D and MS method 2 in negative mode


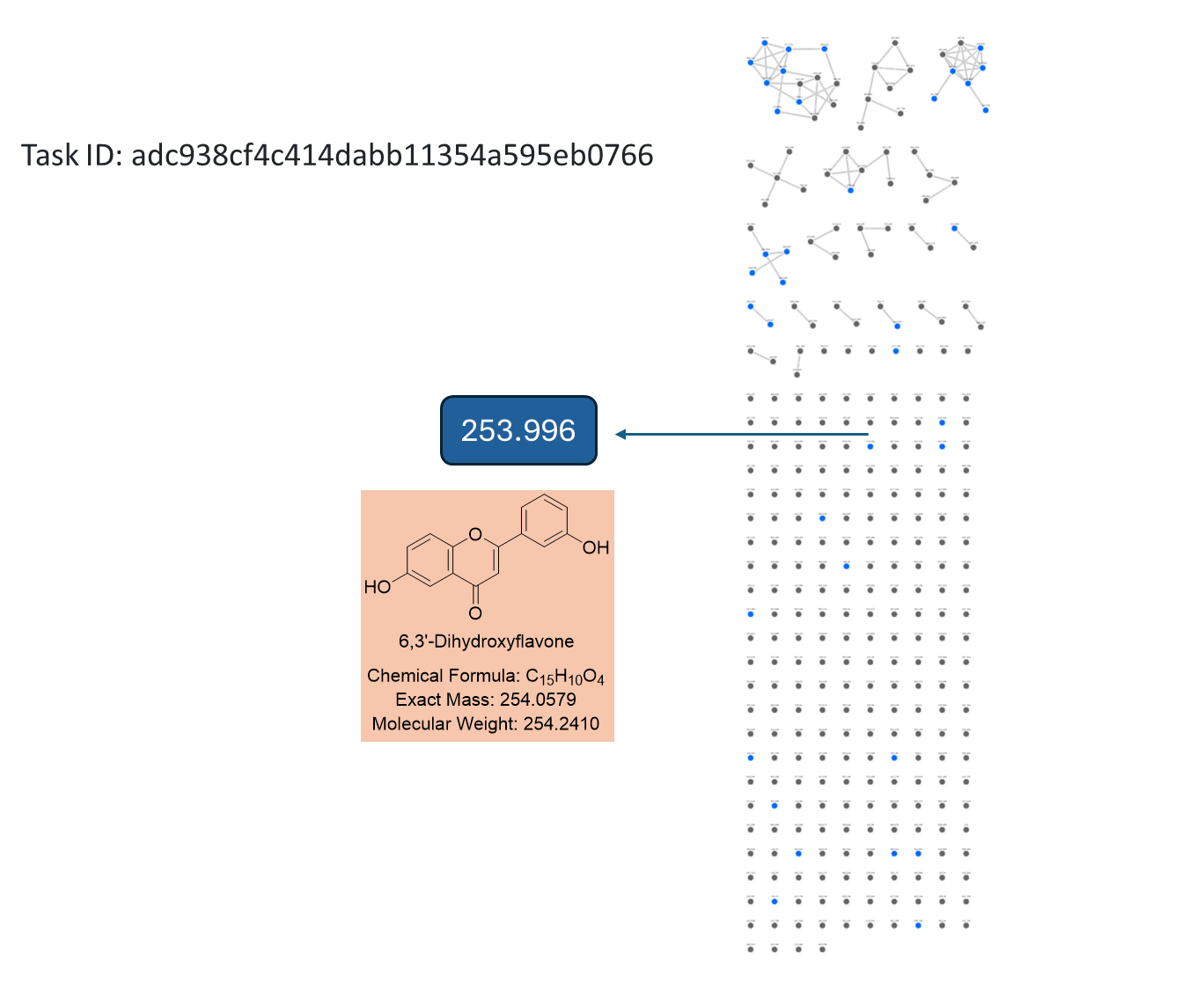


**S15**: Extracted ion chromatogram and MS/MS spectral comparison (experimental vs. library) for 6, 3’-dihydroxyflavone


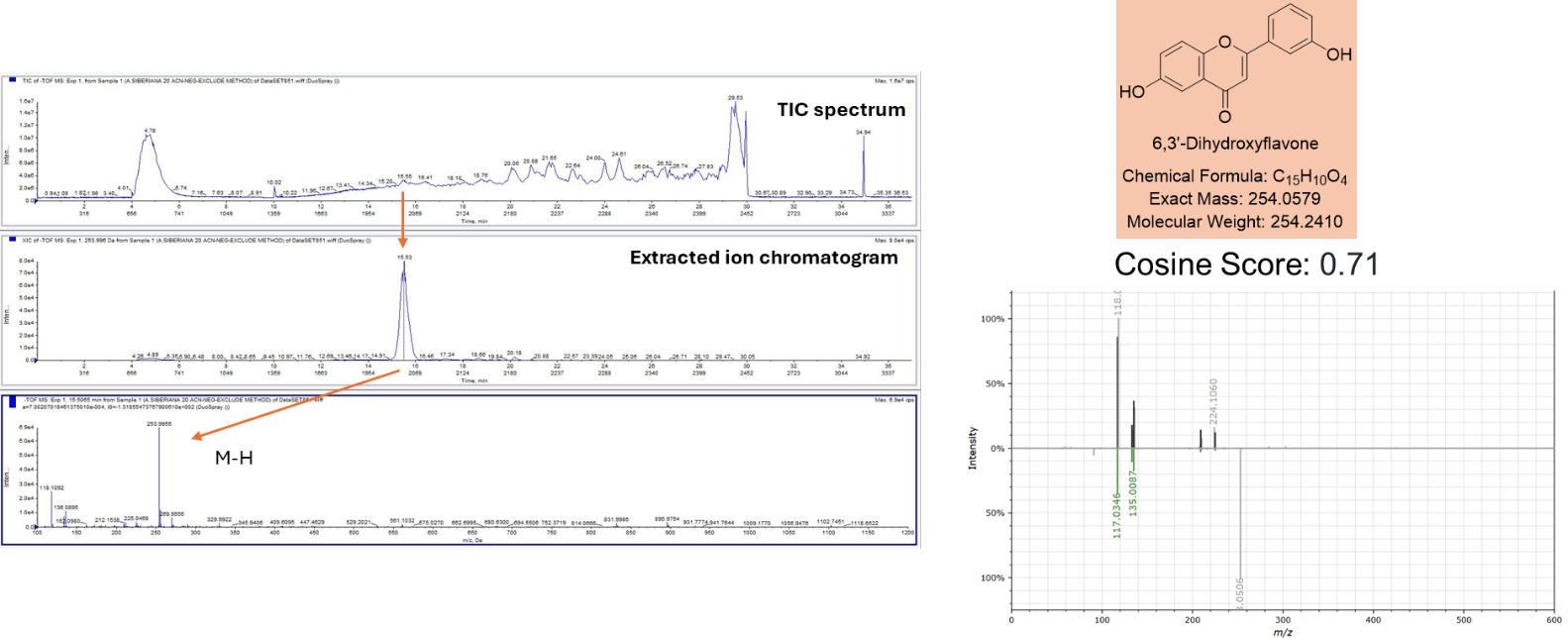

Supplement: Supporting Information — Additional supporting information can be found online in the Supporting Information section. [file 1244498.f1.docx]
